# Supplementary material for: Lower novelty-related locus coeruleus function is associated with Aβ-related cognitive decline in clinically healthy individuals
Source: Nat Commun. 2022 Mar 23;13:1571. doi: 10.1038/s41467-022-28986-2 (PMC8943159; doi:10.1038/s41467-022-28986-2)
Supplement: Supplementary file 1 — Supplementary Information [file 41467_2022_28986_MOESM1_ESM.pdf]

SUPPLEMENTARY MATERIAL

Lower novelty-related locus coeruleus function is associated with Aβ-related cognitive decline in clinically healthy individuals

Authors: Prokopis C. Prokopiou[1†], Nina Engels-Domínguez[1,2†], Kathryn V. Papp[3,4], Matthew R. Scott[4,5], Aaron P. Schultz[4,6], Christoph Schneider[1], Michelle E. Farrell[4], Rachel F. Buckley[3,4,7], Yakeel T. Quiroz[4,8], Georges El Fakhri[1], Dorene M. Rentz[3,4], Reisa A. Sperling[3,4], Keith A. Johnson[1,3,4], Heidi I.L. Jacobs[1,2*]

† Each of these authors contributed equally

Affiliations:

[1] Gordon Center for Medical Imaging, Department of Radiology, Massachusetts General Hospital, Harvard Medical School, Boston, MA, USA.

[2] Faculty of Health, Medicine and Life Sciences, School for Mental Health and Neuroscience, Alzheimer Centre Limburg, Maastricht University, Maastricht, The Netherlands.

[3] Center for Alzheimer Research and Treatment, Department of Neurology, Brigham and Women's Hospital, Harvard Medical School, Boston, MA, USA.

[4] Department of Neurology, Massachusetts General Hospital, Harvard Medical School, Boston, MA, USA.

[5] Department of Biostatistics, Boston University, Boston, MA, USA.

[6] The Athinoula A. Martinos Center for Biomedical Imaging, Department of Radiology, Massachusetts General Hospital, Harvard Medical School, Boston, MA, USA.

[7] Melbourne School of Psychological Sciences, University of Melbourne, Victoria, Australia

[8] Department of Psychiatry, Massachusetts General Hospital, Harvard Medical School, Boston, MA, USA.

* To whom correspondence should be addressed: Dr. Heidi Jacobs, PhD, Department of Radiology, Massachusetts General Hospital/Harvard Medical School, Boston MA 02114, USA, Email: hjacobs@mgh.harvard.edu

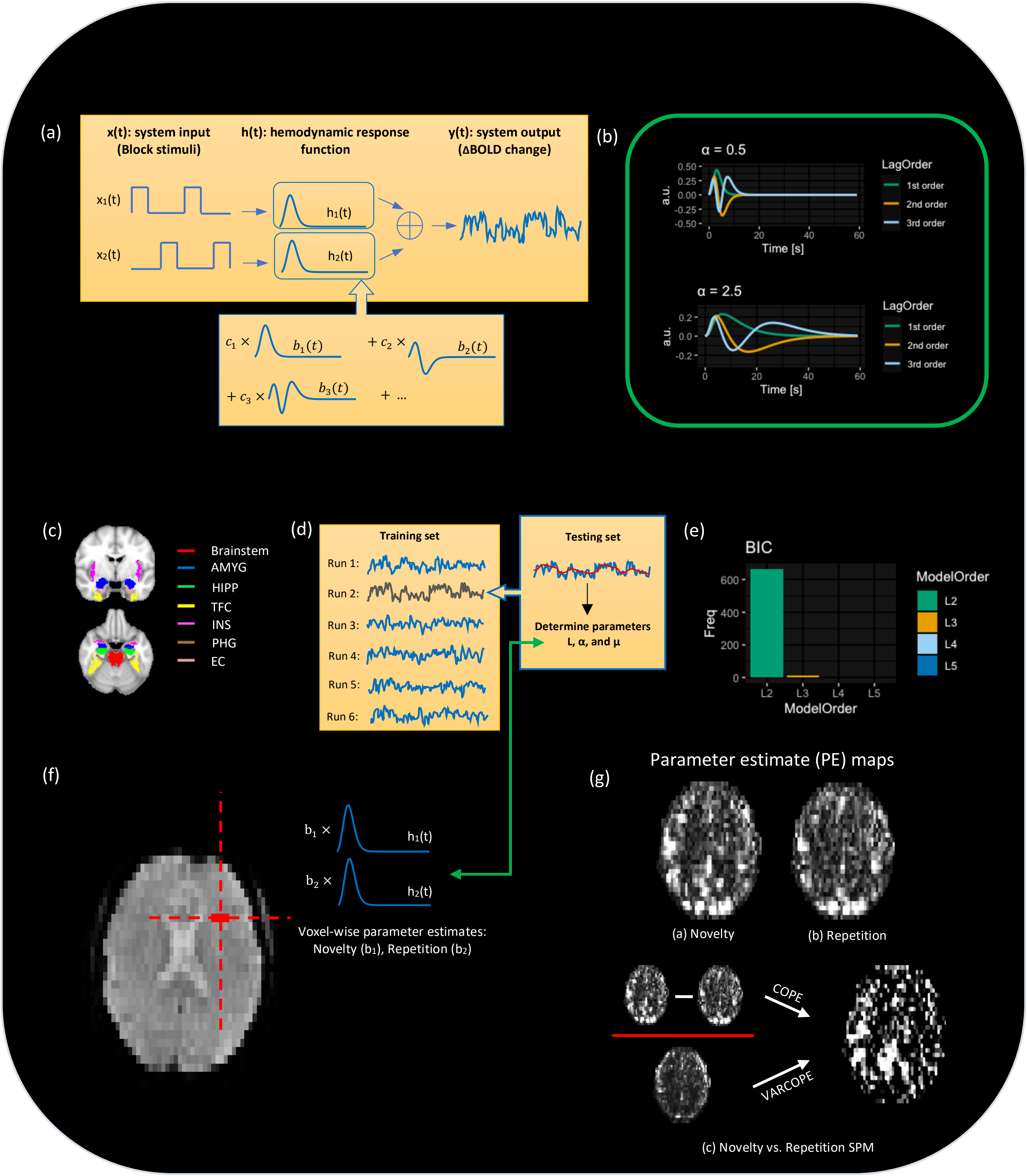

(a)
x(t): system input
(Block stimuli)
h(t): hemodynamic response function
y(t): system output
(ΔBOLD change)
x1(t)
x2(t)
h1(t)
h2(t)
c1 × b1(t) + c2 × b2(t)
+ c3 × b3(t) + ...
(b)
α = 0.5
α = 2.5
LagOrder
1st order
2nd order
3rd order
a.u.
Time [s]
(c)
Brainstem
AMYG
HIPP
TFC
INS
PHG
EC
(d)
Training set
Run 1:
Run 2:
Run 3:
Run 4:
Run 5:
Run 6:
Testing set
Determine parameters
L, α, and μ
(e)
BIC
Freq
ModelOrder
L2
L3
L4
L5
(f)
b1 × h1(t)
b2 × h2(t)
Voxel-wise parameter estimates:
Novelty (b1), Repetition (b2)
(g)
Parameter estimate (PE) maps
(a) Novelty
(b) Repetition
COPE
VARCOPE
(c) Novelty vs. Repetition SPM

Supplementary Fig. 1: Schematic diagram of BOLD-fMRI data processing. (a) The BOLD-fMRI signal was modelled as the sum of the output of two parallel block-cascade linear FIR systems, with each block corresponding to a different experimental condition. Each subsystem is fully characterized by its associated HRF. The unknown HRFs were estimated directly from the data using function expansions along with the spherical Laguerre basis functions (see Methods). (b) The first 3 spherical Laguerre functions obtained using α=0.5 (top panel) and α=2.5 (top panel). Smaller α values correspond to HRFs with faster dynamics. Larger α values correspond to HRF estimates with slower dynamics. (c) The preselected task-relevant ROIs used in this work to study Novelty-related activity and LC-FC. In the analysis of LC-FC the brainstem ROI was excluded. (d) Schematic diagram of the 6-fold cross-validation scheme used to obtain optimal values for the unknown parameters of the spherical Laguerre model (L, α, μ). HRF estimation was initially performed in the ROIs. The BOLD-fMRI time-series of each run were sequentially used as testing set and the time-series of the remaining 5 segments were used as training set. At each iteration, model parameter estimates were obtained using the training set and model performance was evaluated using the testing set in terms of the model generalization mean squared error. (e) Histogram of BIC values obtained across all subjects, ROIs, and runs. The histogram suggests that a second order spherical Laguerre model (i.e. L=2) is sufficient to describe the underlying dynamics of the HRF associated with each experimental condition. (f) The Laguerre model parameters L, α and μ defined in the ROI analysis as described in (d) were fixed and used to obtain voxel-wise condition-dependent HRF estimates. Subsequently, the voxel-specific HRF estimates were convoluted with their associated condition-dependent block-timeseries and used in a GLM analysis to obtain parameter estimates (PEs; beta coefficients) quantifying the strength of the BOLD signal response to each experimental condition. (g) The estimated PEs were used to construct contrast (COPE) images and to obtain statistical parameter maps (SPM) of brain activity in response to NvR. Abbreviations: amygdala (AMYG), Bayesian information criterion (BIC), contrast of parameter estimate (COPE), blood oxygenation level-dependent (BOLD), entorhinal cortex (EC), hippocampus (HIPP), insula (INS), parahippocampal gyrus (PHG), temporal fusiform cortex (TFC) and variance of contrast of parameter estimate (VARCOPE).

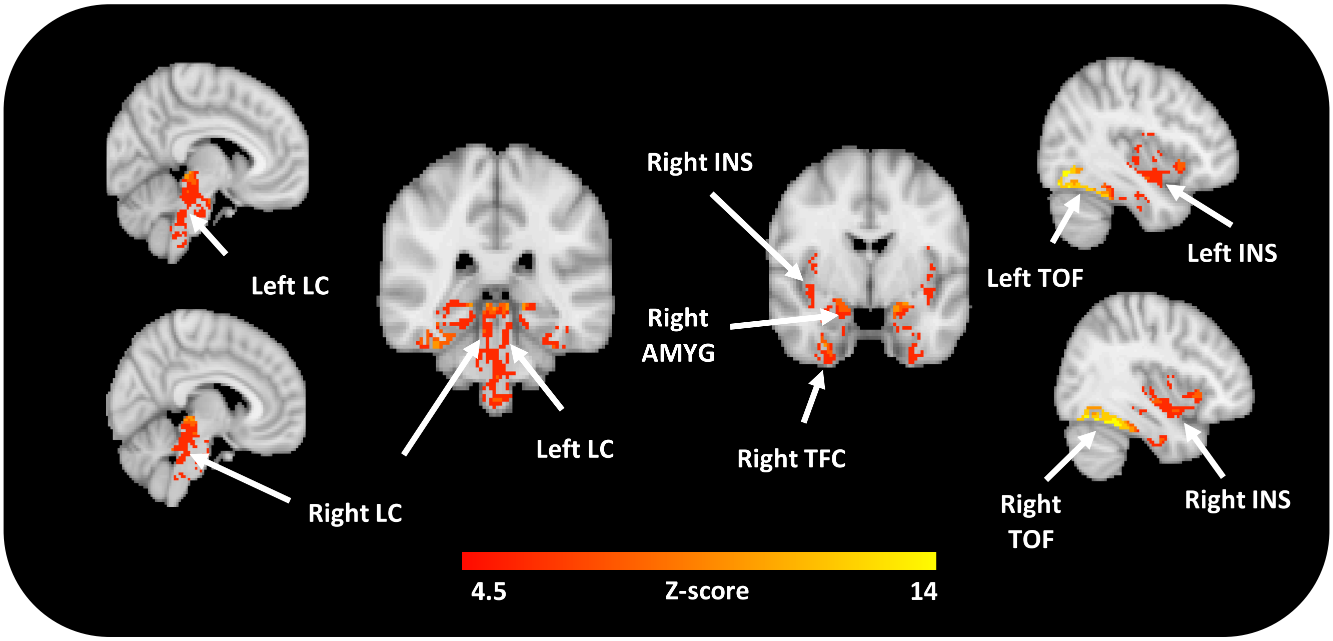

Supplementary Fig. 2: Validation results for voxel-wise analysis of brain activity in predefined regions of interest during Novelty versus Repetition using unsmoothed data. This analysis was performed to demonstrate the robustness of our main findings (Fig. 3a) at a higher spatial resolution (voxel volume ~48.05 mm^3), which is considerably higher compared to studies [1, 2, 3, 4, 5] with a spherical smoothing kernel of 6 or 8 mm (FWHM; see Supplementary results S1). Inference was performed using mixed-effects models including NvR contrast estimates as outcome variable, age and sex as fixed effects, random intercepts for participants and slopes for fMRI runs. The brain activation maps were corrected for multiple comparisons using cluster-extent based thresholding (number of participants n = 128; cluster defining threshold $Z > 4.5$, two-tailed $p < 0.05$, family-wise error (FWER) corrected). Abbreviations: amygdala (AMYG), insula (INS), locus coeruleus (LC), temporal fusiform cortex (TFC) and temporal occipital fusiform cortex (TOF).

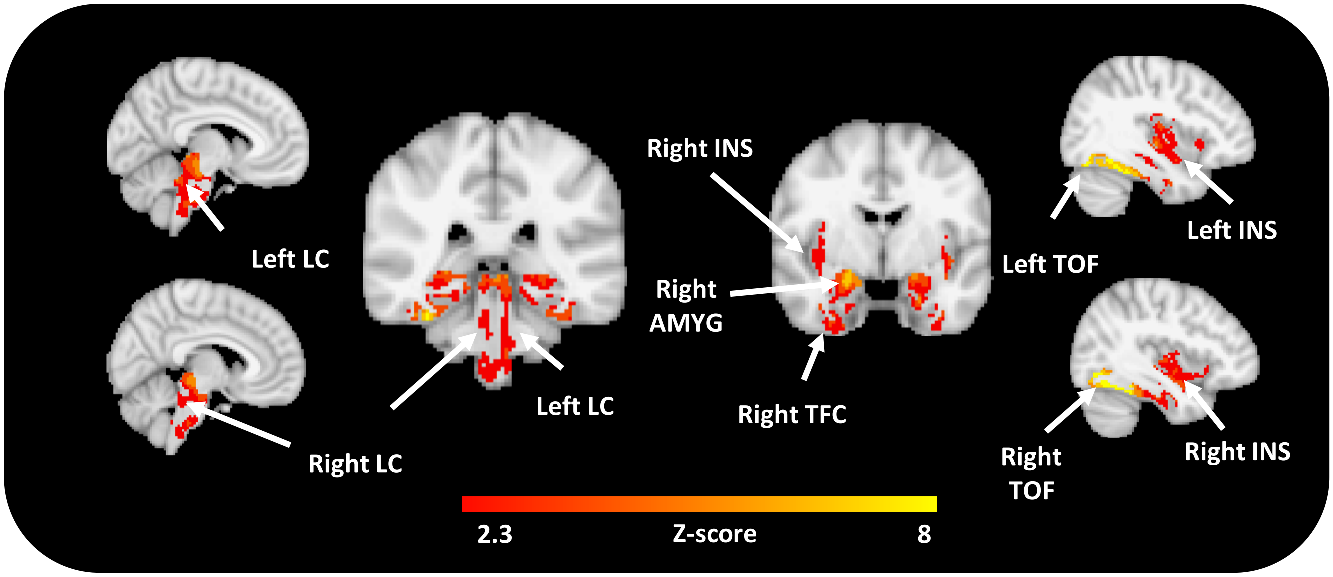

Supplementary Fig. 3: Validation results for voxel-wise analysis of brain activity in predefined regions of interest during Novelty versus Repetition using the Replication Dataset. The details on this dataset are provided in the Participants subsection in the manuscript, and demographic characteristics in Supplementary Table 1. Inference was performed using mixed-effects models including NvR contrast estimates as outcome variable, age and sex as fixed effects, random intercepts for participants and slopes for fMRI runs. The brain activation maps were corrected for multiple comparisons using cluster-extent based thresholding (number of participants $n = 41$; cluster defining threshold $Z > 2.3$, two-tailed $p < 0.05$, family-wise error (FWER) corrected). It should be noted that the size of the Replication Dataset is reduced by 68% compared to the original dataset that was used in our study, which explains the overall decrease in effect size compared to the results shown in Fig. 3. Abbreviations: amygdala (AMYG), insula (INS), locus coeruleus (LC), temporal fusiform cortex (TFC) and temporal occipital fusiform cortex (TOF).

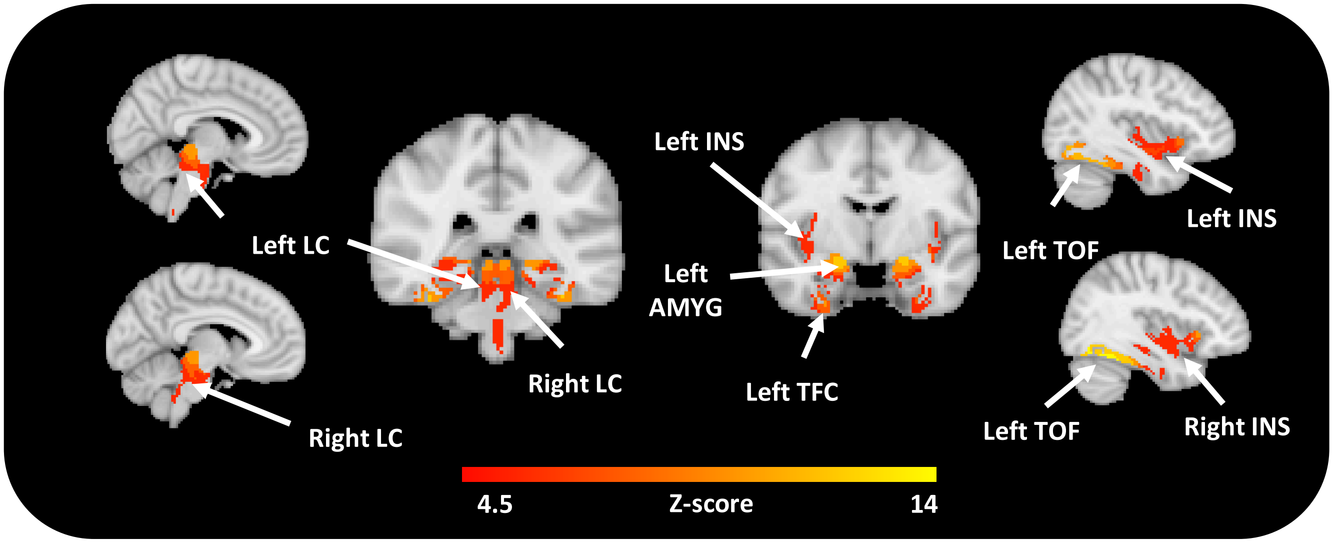

Supplementary Fig. 4: Validation results for voxel-wise analysis of brain activity in predefined regions of interest during Novelty versus Repetition using grey matter density as a voxel-wise covariate. Inference was performed using mixed-effects models including NvR contrast estimates as outcome variable, age and sex as fixed effects, random intercepts for participants and slopes for fMRI runs. The brain activation maps were corrected for multiple comparisons using cluster-extent based thresholding (number of participants $n = 128$; cluster defining threshold $Z > 4.5$, two-tailed $p < 0.05$, family-wise error (FWER) corrected). Abbreviations: amygdala (AMYG), hippocampus (HIPP), insula (INS), locus coeruleus (LC), temporal fusiform cortex (TFC) and temporal occipital fusiform cortex (TOF).

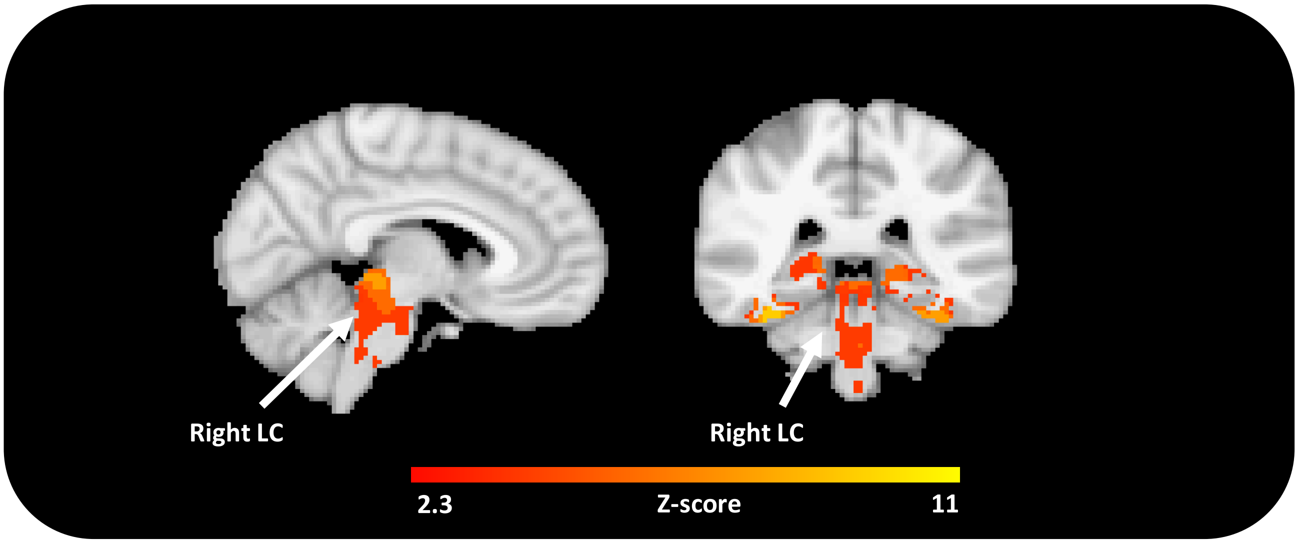

Supplementary Fig. 5: Validation results for voxel-wise analysis of brain activity in predefined regions of interest during Novelty versus Repetition using the Matched Dataset that includes 36 Aβ+ and 36 Aβ- individuals of the original cohort with approximately equal distributions of age, sex and years of education. The selection of the Aβ- individuals was performed using propensity-based matching. Inference was performed using mixed-effects models including NvR contrast estimates as outcome variable, age and sex as fixed effects, random intercepts for participants and slopes for fMRI runs. The brain activation maps were corrected for multiple comparisons using cluster-extent based thresholding (number of participants $n = 72$; cluster defining threshold $Z > 2.3$, two-tailed $p < 0.05$, family-wise error (FWER) corrected). Abbreviation: locus coeruleus (LC).

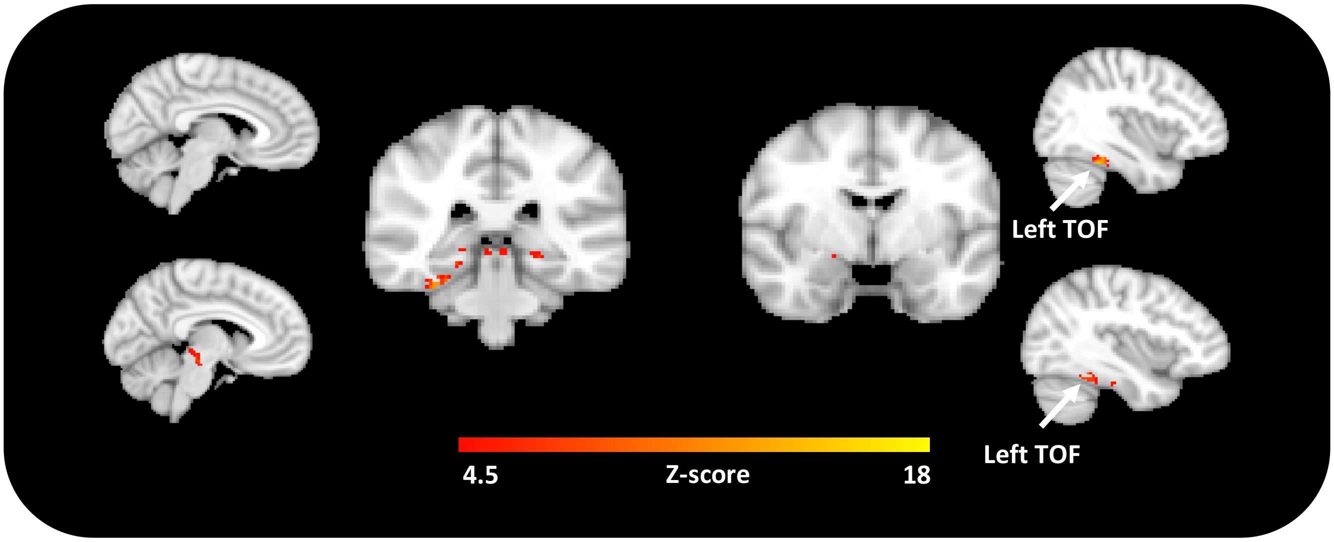

Supplementary Fig. 6: Voxel-wise analysis of brain activity in predefined regions of interest during Novelty versus Fixation. Brain activation maps obtained during Novel face-name stimuli versus Fixation: greater activation was observed during NvF of voxels within the bilateral temporal occipital fusiform cortex. Inference was performed using mixed-effects models including NvF contrast estimates as outcome variable, age and sex as fixed effects, random intercepts for participants and slopes for fMRI runs. The brain activation maps were corrected for multiple comparisons using cluster-extent based thresholding (number of participants $n = 128$; cluster defining threshold $Z > 4.5$, two-tailed $p < 0.05$, family-wise error (FWER) corrected). In addition, greater deactivation was observed during Repeated face-name stimuli versus Fixation of voxels within the medial temporal lobe, a phenomenon also known as repetition suppression, in agreement with previous studies in the literature [91, 92, 93]. However, none of these deactivated brain regions for RvF survived cluster-based correction for multiple comparisons. Abbreviation: temporal occipital fusiform cortex (TOF).

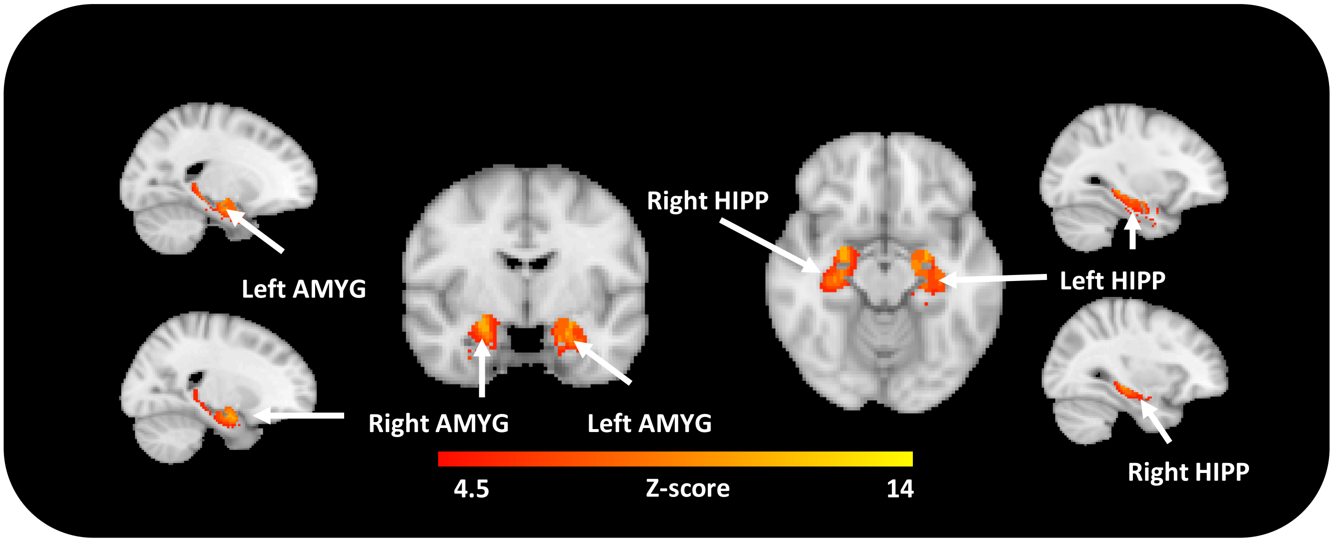

Supplementary Fig. 7: Validation results for voxel-wise analysis of LC functional connectivity in predefined regions of interest during Novelty versus Repetition using a physiological regressor extracted from an eroded version of the original LC ROI (Supplementary Fig. 8 - blue ROI). Inference was performed using mixed-effects models including NvR LC-FC contrast estimates as outcome variable, age and sex as fixed effects, random intercepts for participants and slopes for fMRI runs. The brain activation maps were corrected for multiple comparisons using cluster-extent based thresholding (number of participants $n = 128$; cluster defining threshold $Z > 4.5$, two-tailed $p < 0.05$, family-wise error (FWER) corrected). Abbreviations: amygdala (AMYG) and hippocampus (HIPP).

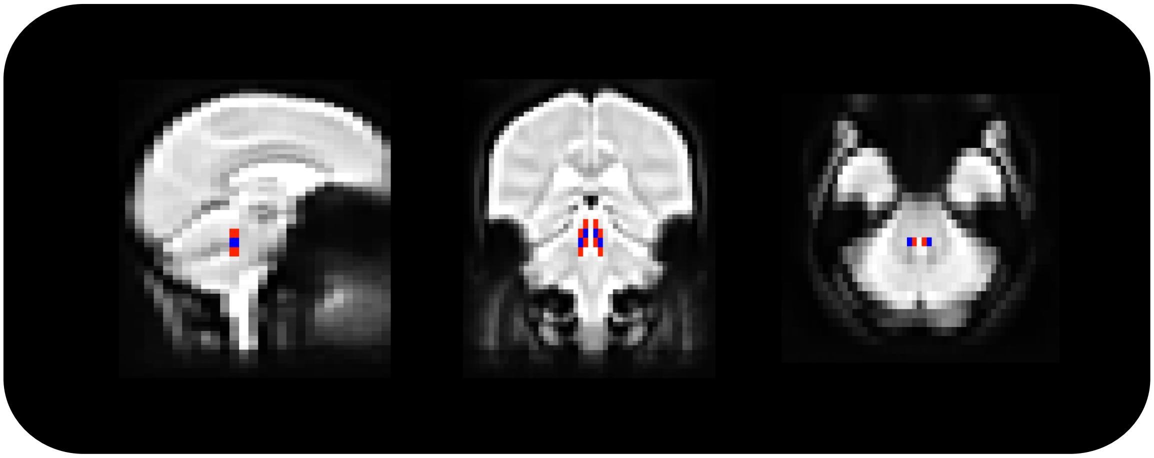

Supplementary Fig. 8: Locus coeruleus ROI and template of the participants' native functional space. The functional template was constructed using the participants' BOLD-fMRI data and the ANTs multivariate template construction tool. A group LC ROI (shown in red) was constructed by first warping an existing postmortem validated template of the LC [24] to the individual T1 image of each participant, and subsequently warping the image of the LC template from their native structural T1 space into the functional template. The group LC ROI was defined as the region where the greatest convergence of LC voxels across participants is observed, and this was back-projected to each native functional space of each participant. The eroded version (60% volume reduction) of the LC ROI (blue) was used to replicate our FC findings using the original LC ROI (red).

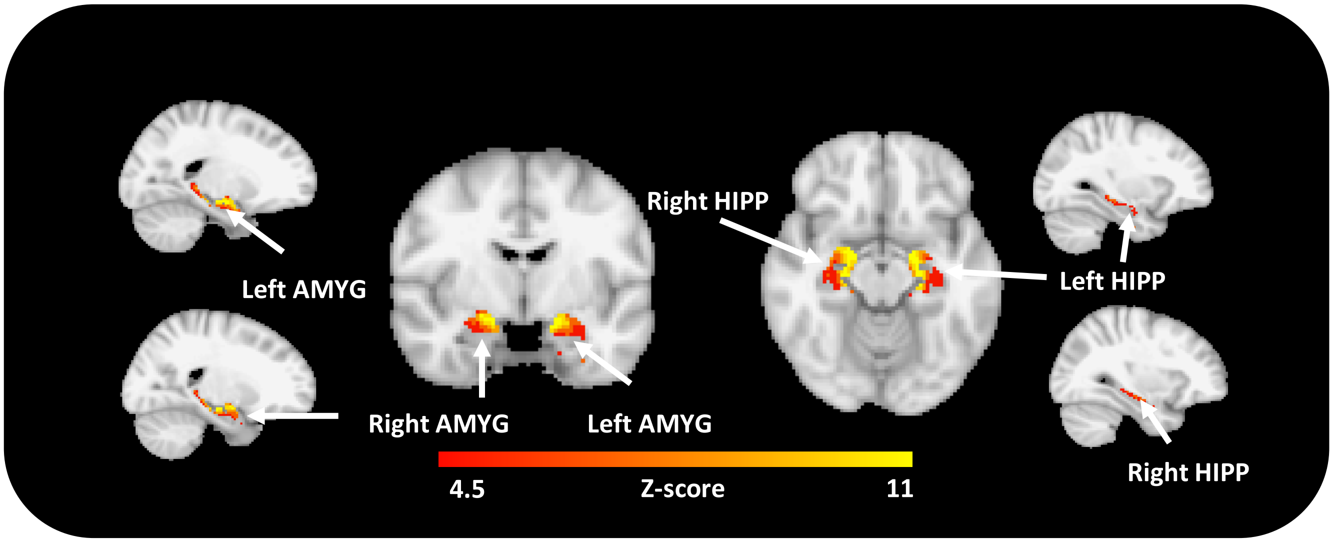

Supplementary Fig. 9: Validation results for voxel-wise analysis of LC functional connectivity in predefined regions of interest during Novelty versus Repetition using unsmoothed data. Inference was performed using mixed-effects models including NvR LC-FC contrast estimates as outcome variable, age and sex as fixed effects, random intercepts for participants and slopes for fMRI runs. The brain activation maps were corrected for multiple comparisons using cluster-extent based thresholding (number of participants n = 128; cluster defining threshold $Z > 4.5$, two-tailed $p < 0.05$, family-wise error (FWER) corrected). Abbreviations: amygdala (AMYG) and hippocampus (HIPP).

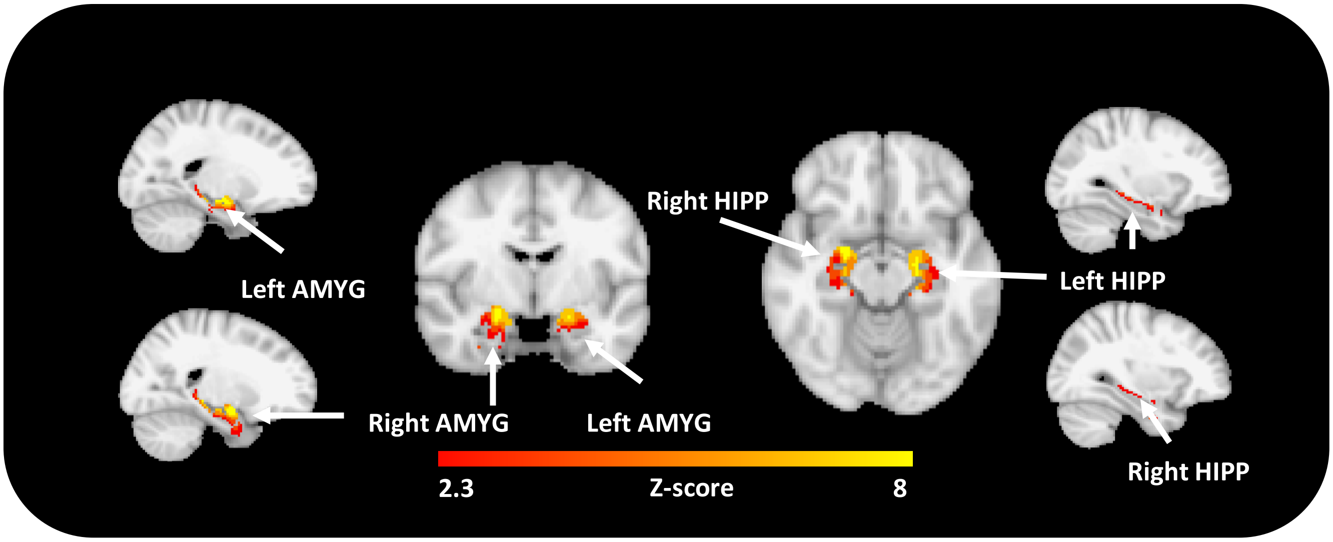

Supplementary Fig. 10: Validation results for voxel-wise analysis of LC functional connectivity in predefined regions of interest during Novelty versus Repetition using the Replication Dataset. The details about this dataset are provided in the Participants subsection in the manuscript, and demographic characteristics in Supplementary Table 1. Inference was performed using mixed-effects models including NvR LC-FC contrast estimates as outcome variable, age and sex as fixed effects, random intercepts for participants and slopes for fMRI runs. The brain activation maps were corrected for multiple comparisons using cluster-extent based thresholding (number of participants $n = 41$; cluster defining threshold $Z > 2.3$, two-tailed $p < 0.05$, family-wise error (FWER) corrected). It should be noted that the size of the Replication Dataset is reduced by 68% compared to the original dataset that was used in our study, which explains the overall decrease in effect size compared to the results shown in Fig. 3. Abbreviations: amygdala (AMYG) and hippocampus (HIPP).

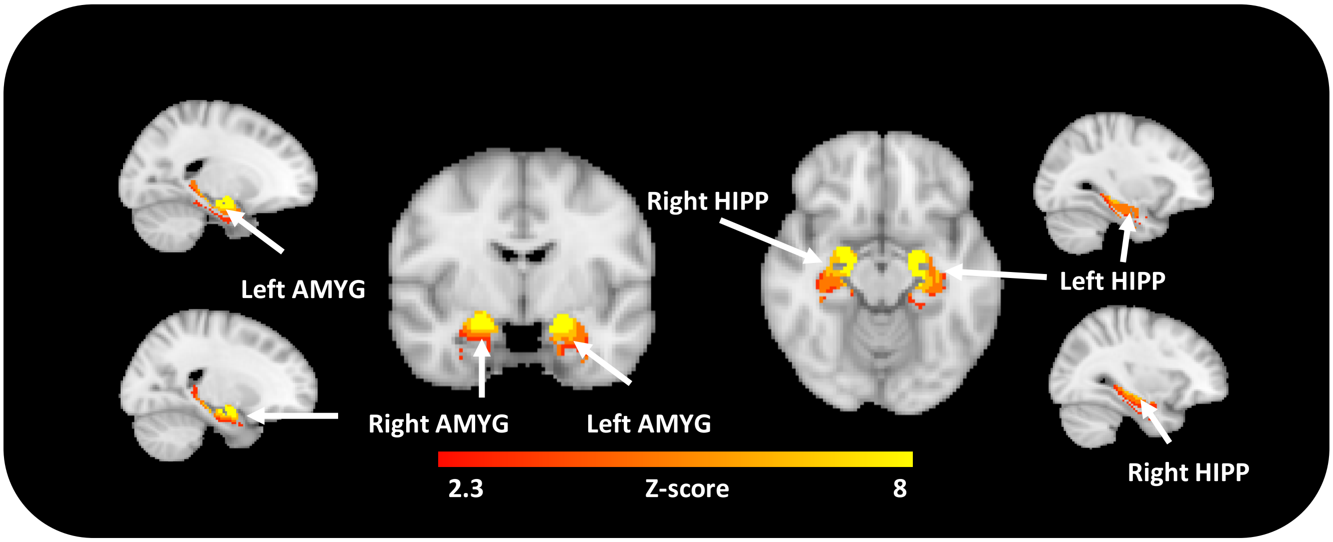

Supplementary Fig. 11: Validation results for voxel-wise analysis of LC functional connectivity in predefined regions of interest during Novelty versus Repetition using the Matched Dataset that includes 36 Aβ+ and 36 Aβ- individuals of the original cohort with approximately equal distributions of age, sex and years of education. The selection of the Aβ- individuals was performed using propensity-based matching. Inference was performed using mixed-effects models including NvR LC-FC contrast estimates as outcome variable, age and sex as fixed effects, random intercepts for participants and slopes for fMRI runs. The FC maps were corrected for multiple comparisons using cluster-extent based thresholding (number of participants $n = 72$; cluster defining threshold $Z > 2.3$, two-tailed $p < 0.05$, family-wise error (FWER) corrected). Abbreviations: amygdala (AMYG) and hippocampus (HIPP).

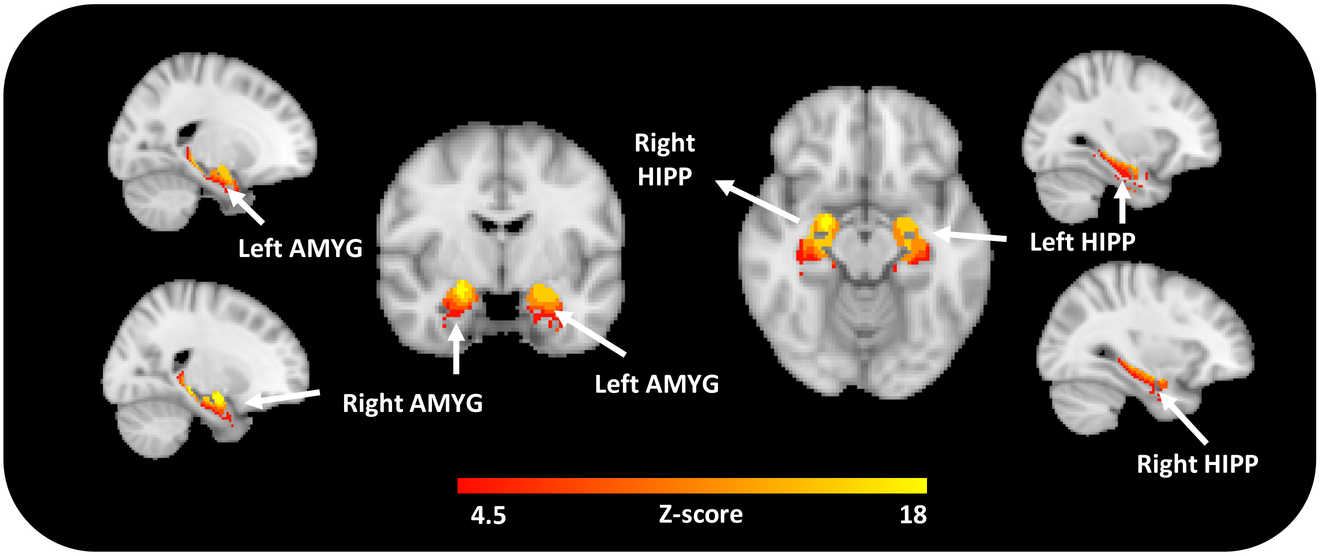

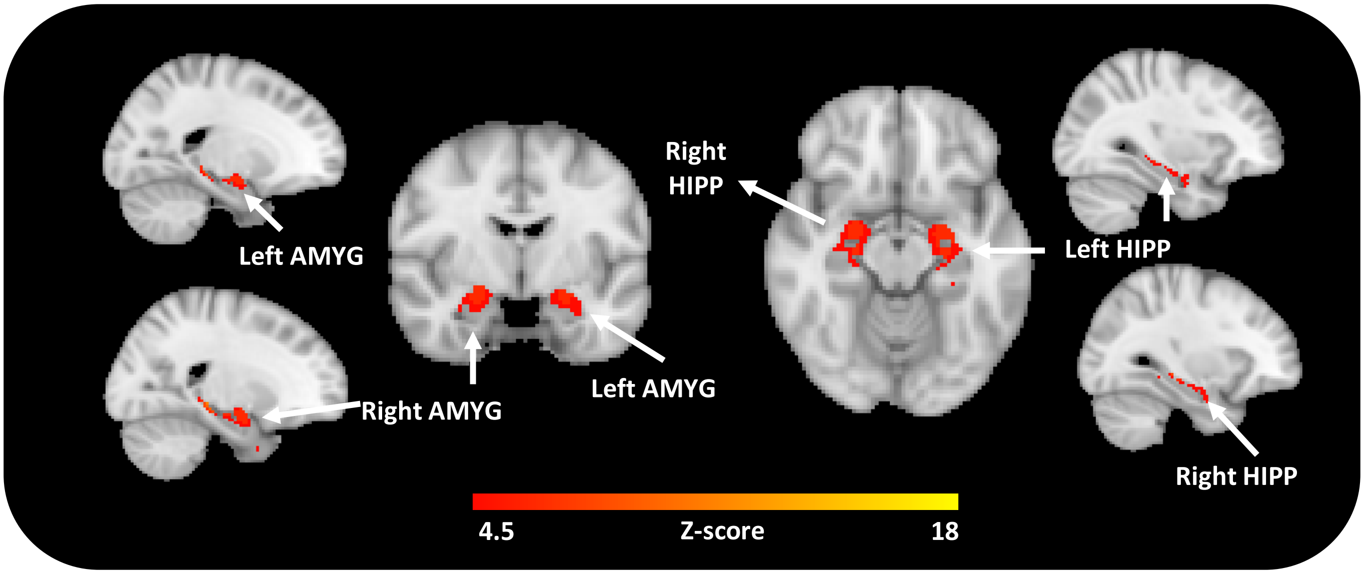

Supplementary Fig. 12: Voxel-wise analyses of LC functional connectivity in predefined regions of interest during Novelty or Repetition versus Fixation. Functional connectivity maps between the LC and the predefined ROIs obtained during NvF or RvF. Top panel: Greater FC was observed during NvF between the LC and voxels within the bilateral amygdala and hippocampus. Bottom panel: Similar patterns of FC between the LC and voxels within the predefined ROIs during RvF were observed. However, the strength of this connectivity was overall weaker relative to NvF. Inference was performed using mixed-effects models including LC-FC contrast estimates as outcome variable, age and sex as fixed effects, random intercepts for participants and slopes for fMRI runs. The FC maps were corrected for multiple comparisons using cluster-extent based thresholding (number of participants $n = 128$; cluster defining threshold $Z > 4.5$, two-tailed $p < 0.05$, family-wise error (FWER) corrected). Abbreviations: amygdala (AMYG) and hippocampus (HIPP).

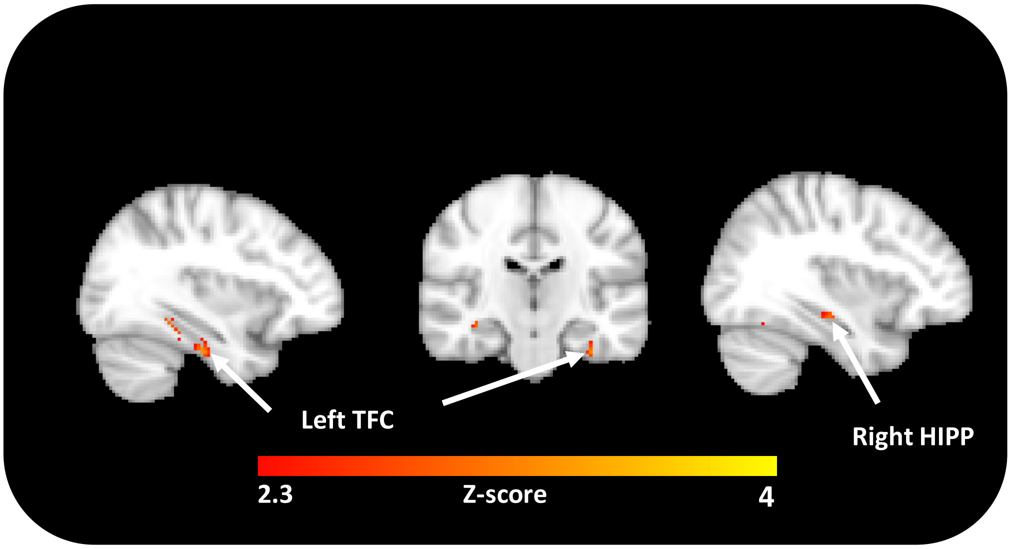

Supplementary Fig. 13: Lower novelty-related brain activity is associated with lower baseline PACC5 performance (FDR-adjusted). Voxel-wise analysis relating NvR activity and cross-sectional PACC5 measurements. The results revealed that lower NvR brain activation in the right hippocampus and left temporal fusiform cortex is associated with lower baseline PACC5 performance. Inference was performed using linear regression including baseline PACC5 as outcome variable, and NvR contrast estimates, age, sex and years of education as predictor variables. The brain activation maps are plotted with a threshold of $Z > 2.3$ and corrected from multiple comparisons using $P_{FDR} < 0.05$ (number of participants $n = 128$). Abbreviations: hippocampus (HIPP) and temporal fusiform cortex (TFC).

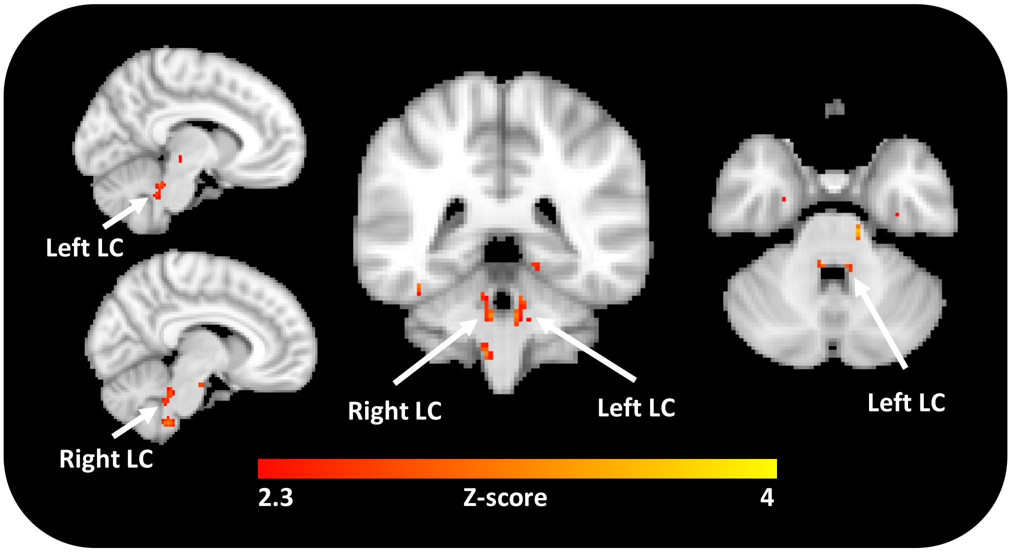

Supplementary Fig. 14: Lower novelty-related LC activity is associated with PACC5 decline (FDR-adjusted). Voxel-wise analysis relating NvR activity and longitudinal PACC5 measurements. Inference was performed using mixed-effects models including PACC5 as outcome variable, NvR contrast estimates, time, their interactions, age, sex and years of education as fixed effects, random intercepts for participants and slopes for time (number of years between baseline and follow-up cognitive assessments). The results revealed that lower NvR bilateral LC activation is associated with greater decline on the PACC5 (see also Fig. 5b). The brain activation maps are plotted with a threshold of $Z > 2.3$ and corrected from multiple comparisons using $P_{FDR} < 0.05$ (number of participants n = 128 and number of observations is 753). Abbreviation: locus coeruleus (LC).

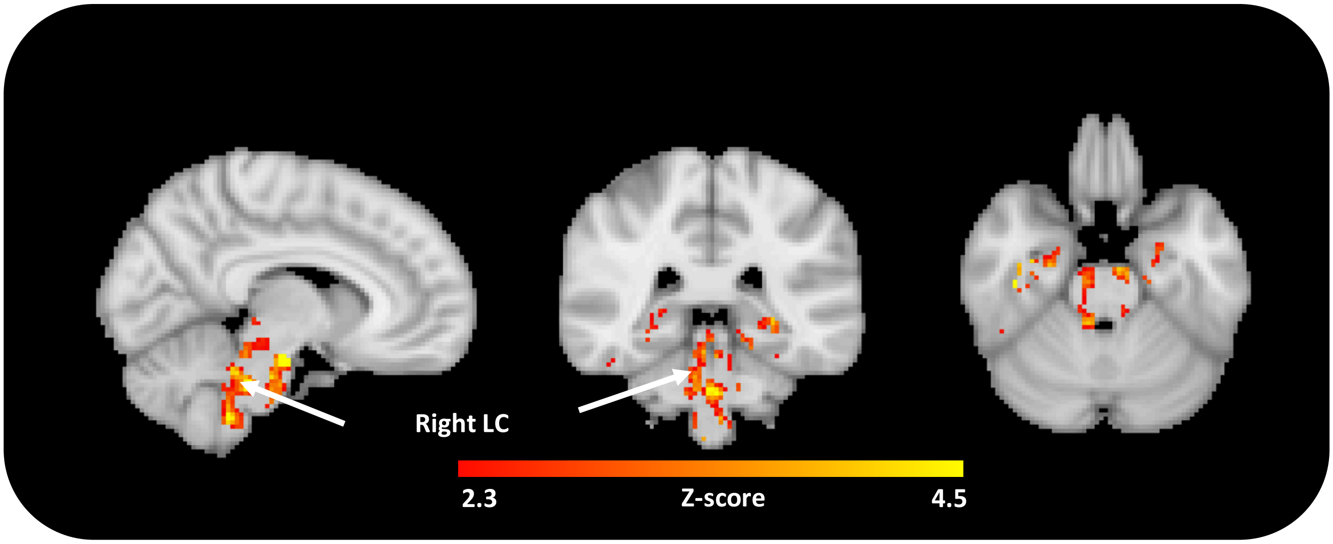

Supplementary Fig. 15: Lower novelty-related LC activity is associated with steeper PACC5 decline when PiB is elevated (FDR-adjusted). Voxel-wise analyses relating NvR activity, PiB and longitudinal PACC5 measurements. Inference was performed using mixed-effects models including PACC5 as outcome variable, NvR contrast estimates, time, PiB, their interactions, age, sex and years of education as fixed effects, random intercepts for participants and slopes for time (number of years between baseline and follow-up cognitive assessments). The brain activation maps are plotted with a threshold of $Z > 2.3$ and corrected from multiple comparisons using $P_{\mathrm{FDR}} < 0.05$ (number of participants $n = 128$). Abbreviation: locus coeruleus (LC).

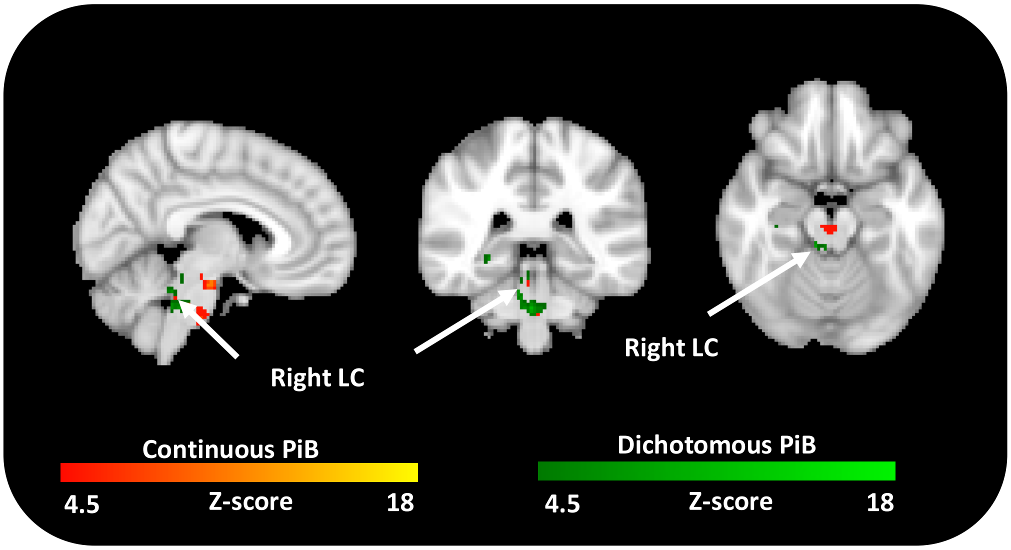

Supplementary Fig. 16: Lower novelty-related LC activity is associated with steeper PACC5 decline when PiB is elevated: comparison of voxel-wise analyses including continuous PiB versus dichotomous PiB. Voxel-wise analyses relating NvR activity, continuous PiB (orange color scale) or dichotomous PiB (PiB status; green color scale), and longitudinal PACC5 measurements. Inference was performed using mixed-effects models including PACC5 as outcome variable, NvR contrast estimates, time, PiB, their interactions, age, sex and years of education as fixed effects, random intercepts for participants and slopes for time (number of years between baseline and follow-up cognitive assessments). The brain activation maps were corrected for multiple comparisons using cluster-extent thresholding (number of participants n = 128 and number of observations is 753; cluster defining threshold $Z > 4.5$, two-tailed $p < 0.05$, family-wise error (FWER) corrected). The results revealed similar brain activation maps obtained using PiB either as a continuous or dichotomous variable. Abbreviations: locus coeruleus (LC) and Pittsburgh Compound-B (PiB).

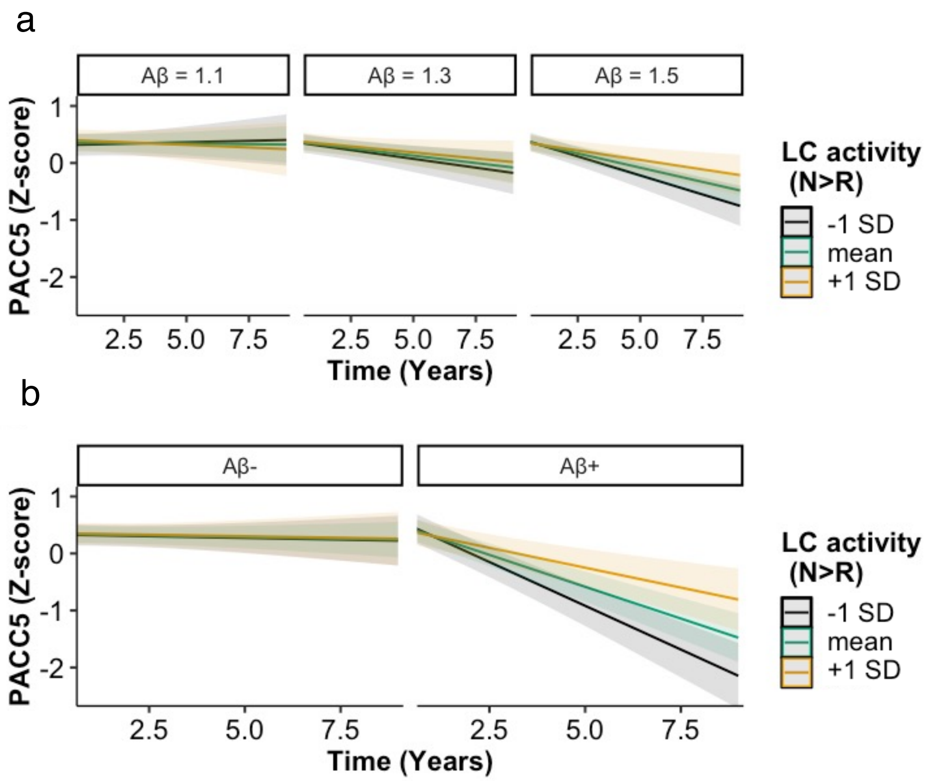

Supplementary Fig. 17: Additional visualization of associations between LC activity during Novelty versus Repetition interacted with PiB and Time, and PACC5. Lower NvR LC activation is associated with steeper cognitive decline in the presence of elevated PiB. (a) Associations between NvR LC activity, PiB and PACC5 over time (number of participants n = 128 and number of observations is 753; B=0.2, $t(621)$=3.37, p<0.001, 95% confidence interval (CI) [0.09, 0.32]). (b) Associations between NvR LC activity, PiB status and PACC5 over time (number of participants n = 128 and number of observations is 753); B=0.14, $t(621)$=2.80, p=0.005, 95% CI[0.022, 0.12]). In all line plots, the estimated marginal mean of the interaction terms is plotted at the mean (green), +1 SD (yellow) and -1 SD (black), but analyses were done continuously. Inference was performed using mixed-effects models including PACC5 as outcome variable, NvR LC activity, time, PiB, their interactions, age, sex and years of education as fixed effects, random intercepts for participants and slopes for time (number of years between baseline and follow-up cognitive assessments). All p-values are two-sided. Shaded areas around the fit lines show 95% CI. Abbreviations: beta-amyloid (Aβ), locus coeruleus (LC), Preclinical Alzheimer Cognitive Composite (PACC5), and Standard Deviation (SD).

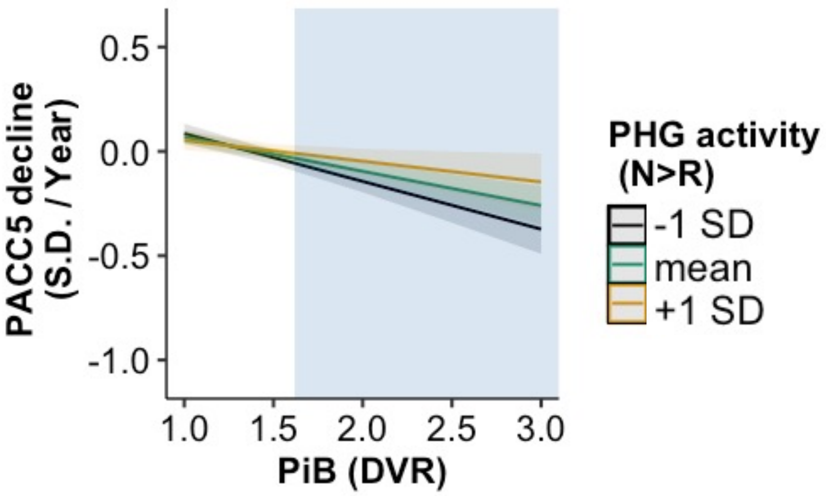

Supplementary Fig. 18: Lower novelty-related right PHG activity is associated with steeper Aβ-related PACC5 decline. Visualization of the interaction between right PHG activity and PiB on PACC5 slopes (number of participants n = 128; B=0.12, $t(121)$=2.34, p=0.02, 95% CI [0.018, 0.22]). The cyan box illustrates the range of PiB values at which NvR PHG activity is associated with PACC5 decline, which corresponds to a DVR value equal to or above 1.62 ($p<0.05$ FDR corrected). The estimated marginal mean of the interaction term is plotted at the mean (green), +1 SD (yellow) and -1 SD (black), but analyses were done continuously. Inference was performed using linear regression including PACC5 decline as outcome variable, and NvR PHG activity, PiB, their interaction, age, sex and years of education as predictor variables. All p-values are two-sided. Shaded areas around the fit lines show 95% CI. Abbreviations: Distribution volume ratio (DVR), parahippocampal gyrus (PHG), Pittsburgh Compound-B (PiB), Preclinical Alzheimer Cognitive Composite (PACC5) and Standard Deviation (SD).

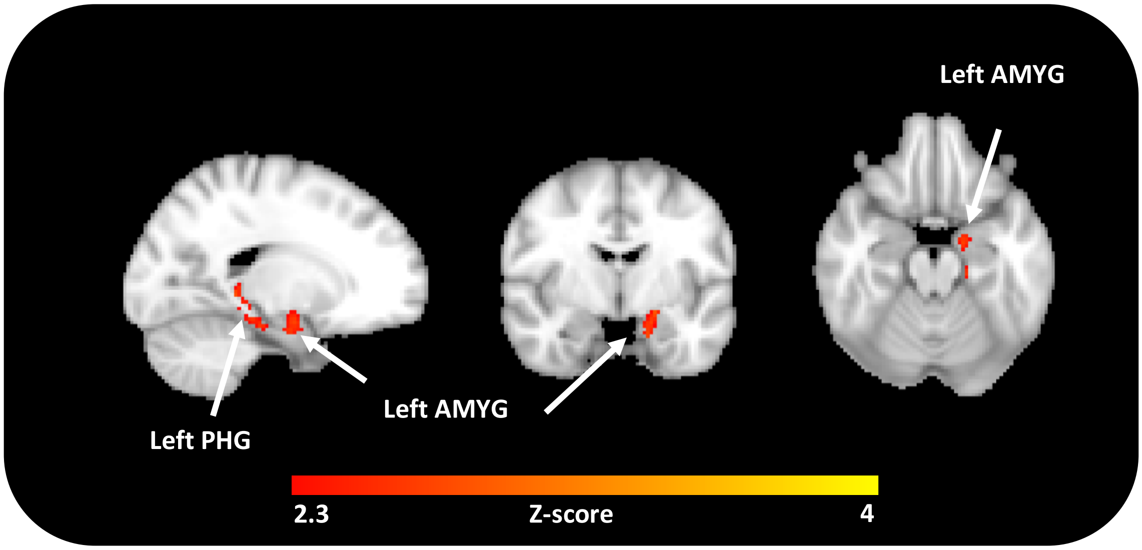

Supplementary Fig. 19: Lower novelty-related LC-MTL functional connectivity is associated with lower baseline PACC5 performance (FDR-adjusted). Voxel-wise analysis relating NvR LC-MTL functional connectivity and cross-sectional PACC5 measurements. The results revealed that lower NvR LC-MTL FC activation is associated with lower baseline PACC5 in the left amygdala and parahippocampal gyrus. Inference was performed using linear regression including baseline PACC5 as outcome variable, and NvR LC-FC contrast estimates, age, sex and years of education as predictor variables. The FC maps are plotted with a threshold of $Z > 2.3$ and corrected for multiple comparisons using $P_{FDR} < 0.05$ (number of participants $n = 128$). Abbreviations: amygdala (AMYG) and parahippocampal gyrus (PHG).

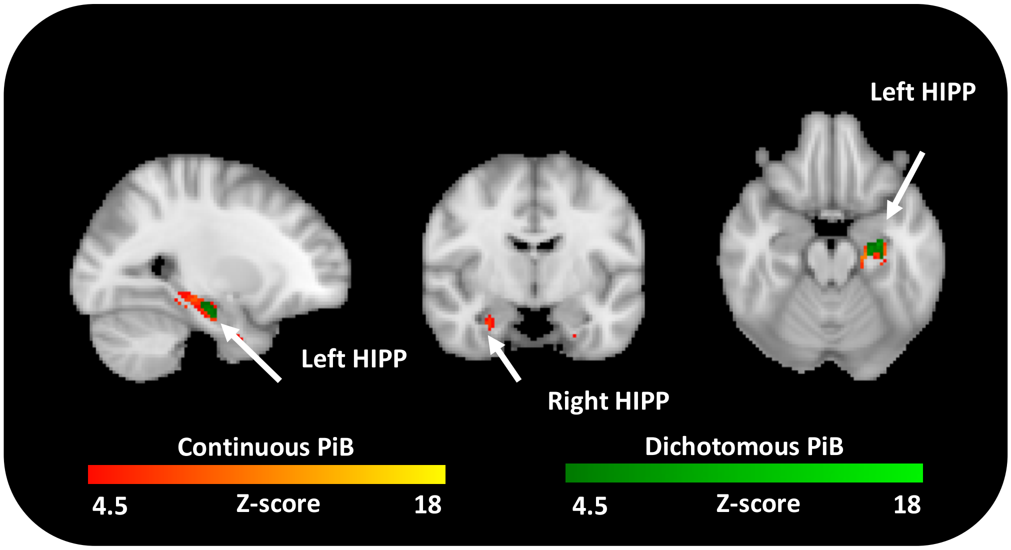

Supplementary Fig. 20: Lower novelty-related FC between the LC and hippocampus is associated with steeper PACC5 decline when PiB is elevated: comparison of voxel-wise analyses including continuous PiB versus dichotomous PiB. Voxel-wise analyses relating NvR LC functional connectivity, continuous PiB (orange color scale) or dichotomous PiB (PiB status; green color scale), and longitudinal PACC5 measurements. Inference was performed using mixed-effects models including PACC5 as outcome variable, NvR LC-FC contrast estimates, time, PiB, their interactions, age, sex and years of education as fixed effects, random intercepts for participants and slopes for time (number of years between baseline and follow-up cognitive assessments). The results revealed concordant FC maps obtained using PiB either as a continuous or dichotomous variable. The FC maps were corrected for multiple comparisons using cluster-extent thresholding (number of participants n = 128 and number of observations is 753; cluster defining threshold $Z > 4.5$, two-tailed $p < 0.05$, family-wise error (FWER) corrected). Abbreviations: hippocampus (HIPP) and Pittsburgh Compound-B (PiB).

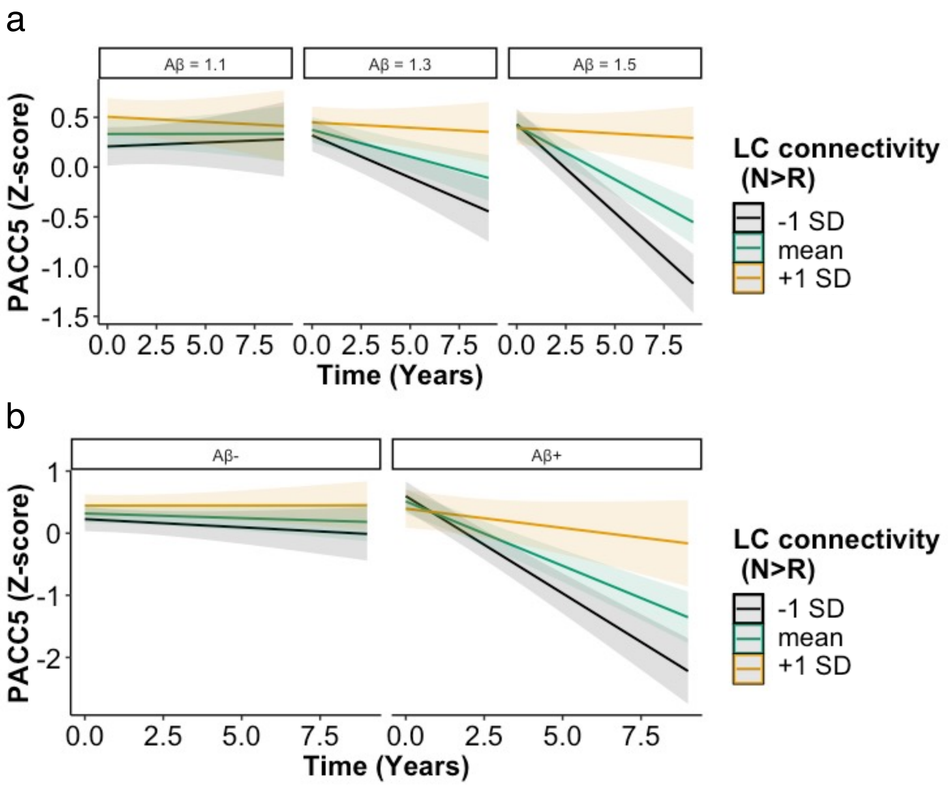

Supplementary Fig. 21: Additional visualization of associations between functional connectivity between the LC and the left hippocampus/parahippocampal gyrus during Novelty versus Repetition interacted with PiB and time, and PACC5 decline. Lower NvR FC between the LC and the left hippocampus and parahippocampal gyrus is associated with steeper cognitive decline in the presence of elevated PiB. (a) Associations between NvR LC FC, PiB and PACC5 over time (number of participants n = 128 and number of observations is 753; B=4.86, $t(621)$=7.45, p<0.001, 95% CI[3.59, 6.13]). (b) Associations between NvR LC FC, PiB status and PACC5 over time (number of observations: n = 128; B=2.36, $t(621)$=3.52, p<0.001, 95% CI[1.06, 3.67]). In all line plots, the estimated marginal mean of the interaction terms is plotted at the mean (green), +1 SD (yellow) and -1 SD (black), but analyses were done continuously. Inference was performed using mixed-effects models including PACC5 as outcome variable, NvR LC-FC activity, time, PiB, their interactions, age, sex and years of education as fixed effects, random intercepts for participants and slopes for time (number of years between baseline and follow-up cognitive assessments). All p-values are two-sided. Shaded areas around the fit lines show 95% CI. Abbreviations: beta-amyloid (Aβ), locus coeruleus (LC), Preclinical Alzheimer Cognitive Composite (PACC5) and Standard Deviation (SD).

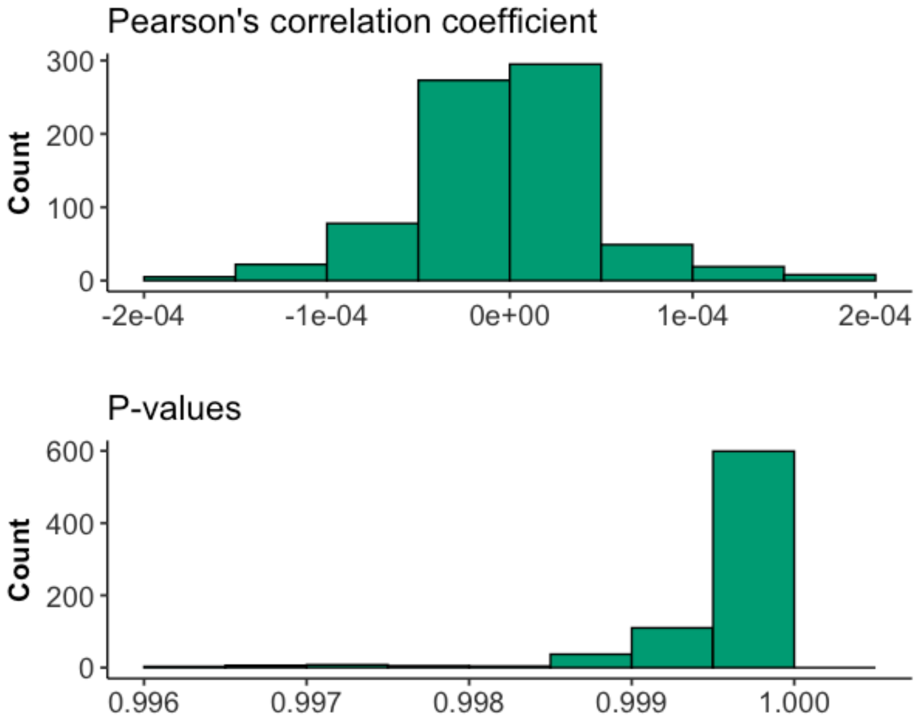

Supplementary Fig. 22: Histogram of pairwise Pearson's correlation coefficients (top) and p-values (bottom) obtained between LC and 4th ventricle ROI time-series (number of time-points N = 127) from all participants and functional runs (number of tests *n* =768). No significant correlations were observed ($r(125)<0.001$, two-tailed $p > 0.9$ uncorrected).

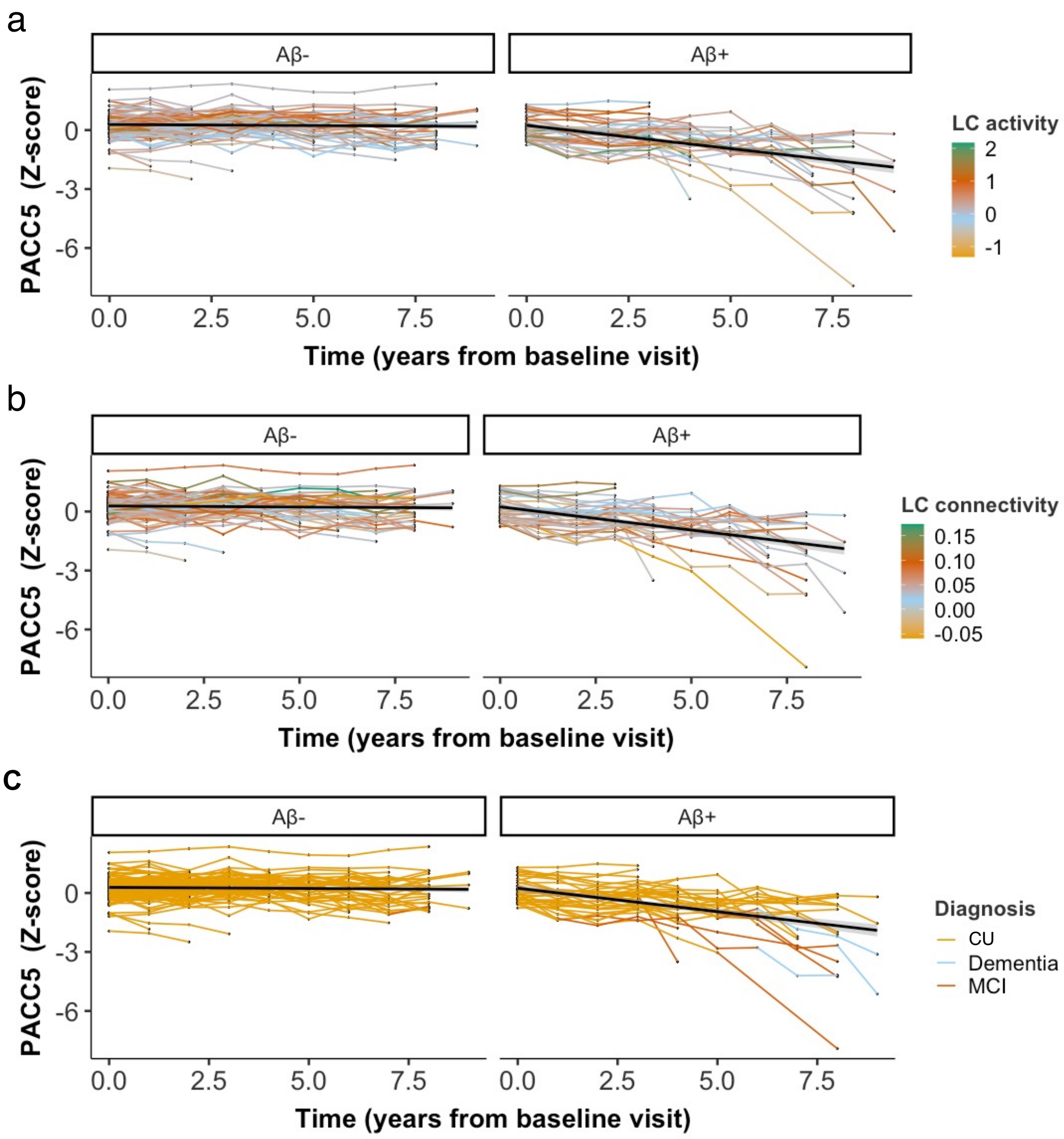

Supplementary Fig. 23: Spaghetti plots of individual PACC5 trajectories with respect to the baseline visit, color coded by magnitude of novelty-related LC activity, LC functional connectivity or clinical disease progression. The color bars indicate (a) baseline NvR LC activity, (b) baseline NvR LC-Left amygdala/hippocampus FC, or (c) consensus diagnosis (cognitively unimpaired (CU), mild cognitive impairment (MCI), or dementia). For each panel the average unadjusted slope is also plotted (black solid lines). Connected dots represent repeated measurements of participants over time. Number of participants *n* = 128 and number of observations is 753. Abbreviation: beta-amyloid (Aβ), cognitively unimpaired (CU), locus coeruleus (LC), mild cognitive impairment (MCI) and Preclinical Alzheimer Cognitive Composite (PACC5).

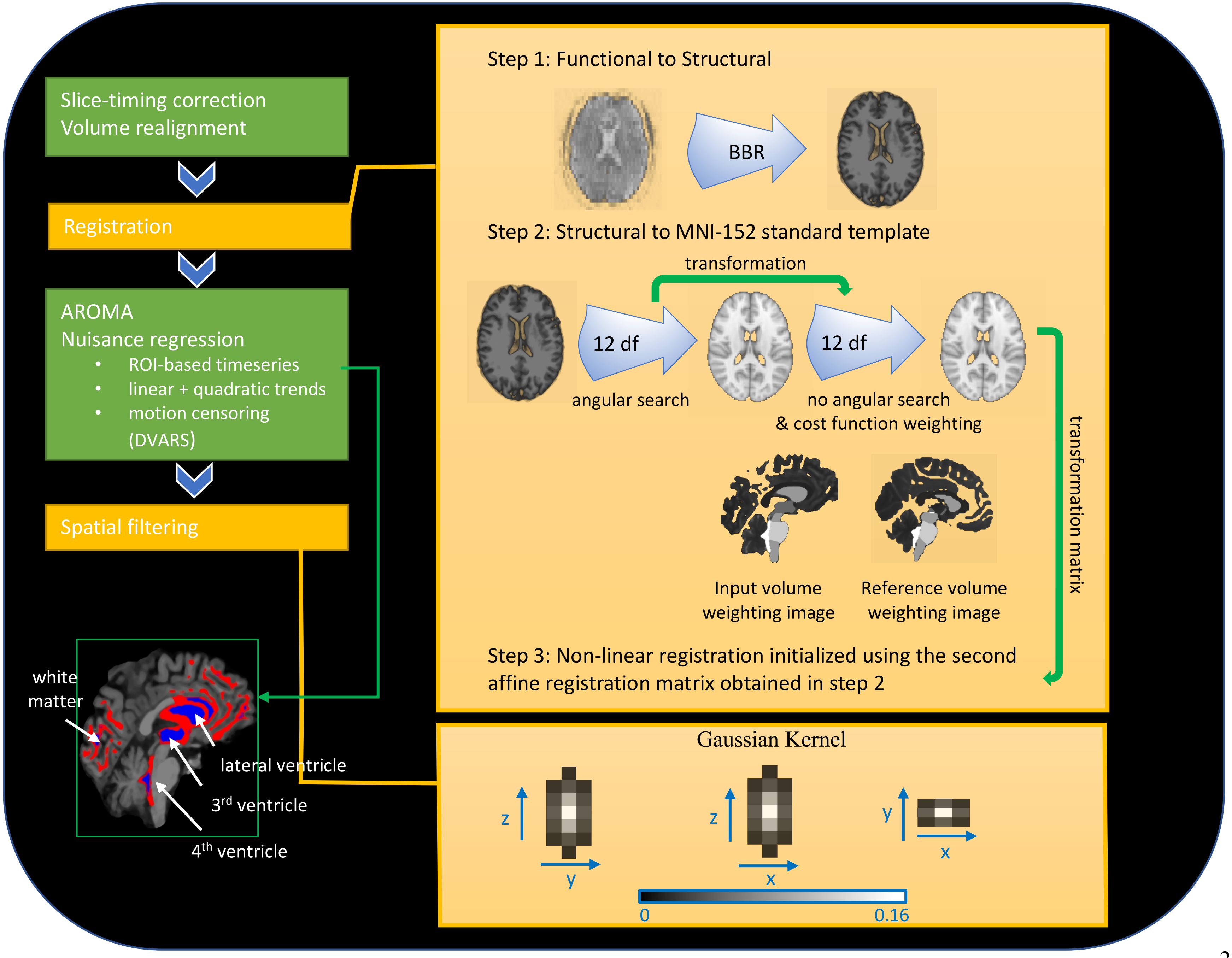
Slice-timing correction
Volume realignment
Registration
AROMA
Nuisance regression
ROI-based timeseries
linear + quadratic trends
motion censoring
(DVARS)
Spatial filtering
white matter
lateral ventricle
3rd ventricle
4th ventricle
Step 1: Functional to Structural
BBR
Step 2: Structural to MNI-152 standard template
transformation
12 df
angular search
12 df
no angular search
& cost function weighting
transformation matrix
Input volume
weighting image
Reference volume
weighting image
Step 3: Non-linear registration initialized using the second affine registration matrix obtained in step 2
Gaussian Kernel
z
y
z
x
y
x
0
0.16

Supplementary Fig. 24: Preprocessing steps of BOLD-fMRI data. We performed slice timing correction, volume realignment, registration to the 2 mm^3 MNI-152 template, motion correction using ICA-AROMA, nuisance regression and spatial filtering. Registration of functional to MNI-152 template volumes was performed in 3 steps. Step 1: the BOLD-fMRI volumes were aligned to the high resolution 1 mm^3 T1 structural image obtained from each subject using boundary-based registration. Step 2: To account for the proximity of the brainstem to the 4th ventricle, its tiny structure and possible partial volume effects, we performed weighted registration of the brainstem. Specifically, the T1 structural image was registered to the MNI-152 template using an affine, linear registration with 12 degrees of freedom. Subsequently, a second affine registration was performed initiated using the transformation matrix obtained from the first affine transformation, as well as cost-function weighting input and reference volumes. In the later affine registration, no angular search was performed. Step 3: a final registration of the T1 structural image to the MNI-152 template was performed using non-linear registration initialed with the second registration matrix obtained in step 2. Spatial filtering was performed using a custom ellipsoid Gaussian kernel stretched towards the z-direction in order to enhance detection of elongated structures within the brainstem. Nuisance regression was performed using the mean ROI time-series, the 6 motion parameters generated during volume realignment, their derivatives and the squares of all the aforementioned time-series. It also included linear and quadratic trends, and additional motion-related regressors defined based on DVARS. Abbreviations: Automatic Removal of Motion Artifacts (AROMA), boundary-based registration (BBR), degrees of freedom (df), derivative of root mean squared variance over voxels (DVARS), Montreal Neurological Institute (MNI) and region of interest (ROI).

n, No	41
Age (years)	75.25 [69.50, 80.75]
Sex, No. (%) = M	19 (46.34)
Education (years)	16.00 [13.0, 18.0]
MMSE (score)	29.00 [29.0, 30.0]
GDS (score)	4.00 [3.0, 8.0]

Supplementary Table 1: Characteristics of Replication Dataset participants. Imaging data of these participants was acquired with a different version of the face-name associative task and four years later compared to the original dataset. Data is presented as medians and [interquartile ranges (IQRS)] for continuous variables and proportions for dichotomous data. Abbreviations: Geriatric Depression Scale (GDS), Male (M) and Mini-Mental State Examination (MMSE).

n, No.	72
Age (years)	76.12 [70.69, 75.66]
Sex, No. (%) = M	36 (50)
Education (years)	16.00 [13.75, 18.00]
MMSE (score)	29.00 [29.00, 30.00]
GDS (score)	2.00 [1.00, 4.00]

Supplementary Table 2: Characteristics of Matched Dataset participants. Participants are matched based on age, sex and years of education at baseline. Data is presented as medians and [interquartile ranges (IQRS)] for continuous variables and proportions for dichotomous data. Abbreviations: Geriatric Depression Scale (GDS), Male (M) and Mini-Mental State Examination (MMSE).

Region	*t*-value	*p*-value	Lower 95% CI	Higher 95% CI
AMYG	12.48	<0.001	2.53	3.48
HIPP	7.73	<0.001	0.85	1.44
PHG	3.26	0.002	0.18	0.75
TFC	19.12	<0.001	5.79	7.13
EC	2.64	0.009	0.09	0.61
INS	3.83	<0.001	0.30	0.95
LC	3.18	0.002	0.19	0.79

Supplementary Table 3: Supplementary statistics for the comparisons of the hemodynamic response function amplitude (HRF peak) obtained within ROIs during Novelty versus Repetition (NvR). The statistical comparisons were performed using a two-tailed paired t-test for $n = 128$. All *p*-values were two-sided and adjusted using FDR. Abbreviations: amygdala (AMYG), condition (COND), confidence interval (CI), entorhinal cortex (EC), hippocampus (HIPP), insula (INS), locus coeruleus (LC), parahippocampal gyrus (PHG) and temporal fusiform cortex (TFC).

Cognitive test	*B*	Standard Error	DF	*t*-value	*p*-value	Lower 95% CI	Upper 95% CI
PACC5	0.05	0.03	623	1.66	0.90	-0.01	0.11
Digit symbol substitution test (DSST)	0.32	0.24	622	1.32	1.0	-0.15	0.79
Free recall element of FCSRT	0.19	0.24	606	0.78	1.0	-0.28	0.66
Total recall element of FCSRT *	0.19	0.05	606	3.71	**<0.001**	0.09	0.30
Delayed recall of the Logical Memory Test	0.09	0.12	622	0.78	1.0	-0.14	0.33
Mini-Mental State Exam (MMSE)	0.10	0.06	623	1.78	0.72	-0.01	0.22
Cognitive Abilities Test (CAT)	0.21	0.20	614	1.03	1.0	-0.19	0.61
Executive Composite	0.01	0.02	619	0.69	1.0	-0.02	0.04
Memory Composite	0.03	0.03	623	1.00	1.0	-0.03	0.09

Supplementary Table 4: Associations between novelty-related LC activity and longitudinal cognitive performance on the subtests of the PACC5, as well as the executive function and memory composite scores. Analyses relating NvR LC activity, time and longitudinal cognitive scores (PACC5, PACC5 subtests, executive function composite score and memory composite score). The analyses were performed using mixed-effects models including PACC5 as outcome variable, NvR LC activity, time, their interaction, age, sex and years of education as fixed effects, random intercepts for participants and slopes for time (number of years between baseline and follow-up cognitive assessments; number of participants $n = 128$ and number of observations is 753). All p-values were two-sided and adjusted using FDR. * Random effects were modeled using only a random intercept for each subject. Reported estimates are unstandardized. Abbreviations: Free and Cued Selective Reminder Test (FCSRT) and Preclinical Alzheimer Cognitive Composite (PACC5).

Cognitive test	*B*	Standard Error	DF	*t*-value	*p*-value	Lower 95% CI	Upper 95% CI
PACC5	0.20	0.06	621	3.37	**<0.001**	0.09	0.32
Digit symbol substitution test (DSST)	1.51	0.52	620	2.92	**<0.001**	0.50	2.51
Free recall element of FCSRT	0.96	0.50	604	1.94	0.45	-0.01	1.93
Total recall element of FCSRT *	0.28	0.12	604	2.31	0.18	0.04	0.52
Delayed recall of the Logical Memory Test	0.24	0.27	620	0.90	1.0	-0.28	0.76
Mini-Mental State Exam (MMSE)	0.32	0.13	621	2.53	0.09	0.07	0.56
Cognitive Abilities Test (CAT)	1.19	0.38	612	3.1	**<0.001**	0.44	1.94
Executive Composite	0.08	0.03	617	2.52	0.09	0.02	0.15
Memory Composite	0.11	0.06	621	1.85	0.07	-0.01	0.23

Supplementary Table 5: Associations between novelty-related LC activity and longitudinal PiB-related cognitive performance on the subtests of the PACC5, as well as executive function and memory scores. Analyses relating NvR LC activity, PiB, time and longitudinal cognitive scores (PACC5, PACC5 subtests, executive function composite score and memory composite score. The analyses were performed using mixed-effects models including PACC5 as outcome variable, NvR LC activity, time, PiB, their interactions, age, sex and years of education as fixed effects, random intercepts for participants and slopes for time (number of years between baseline and follow-up cognitive assessments; number of participants n = 128 and number of observations is 753). All p-values were two-sided and adjusted using FDR. * Random effects were modeled using only a random intercept for each subject. Reported estimates are unstandardized. Abbreviations: Free and Cued Selective Reminder Test (FCSRT) and Preclinical Alzheimer Cognitive Composite (PACC5).

Cognitive test	*B*	Standard Error	DF	*t*-value	*p*-value	Lower 95%CI	Upper 95%CI
PACC5	1.25	0.36	623	3.42	**<0.001**	0.53	1.96
Digit symbol substitution test (DSST)	9.64	2.82	622	3.42	**<0.001**	4.12	15.15
Free recall element of FCSRT	6.29	2.88	606	2.18	0.27	0.66	11.92
Total recall element of FCSRT *	2.10	0.62	606	3.38	**<0.001**	0.88	3.31
Delayed recall of the Logical Memory Test	2.83	1.42	622	2.00	0.45	0.06	5.61
Mini-Mental State Exam (MMSE)	2.33	0.70	623	3.32	**<0.001**	0.96	3.70
Cognitive Abilities Test (CAT)	3.00	2.44	614	1.23	1.0	-1.77	7.78
Executive Composite	0.25	0.19	619	1.31	1.0	-0.12	0.62
Memory Composite	0.81	0.36	623	2.25	0.18	0.11	1.52

Supplementary Table 6: Associations between novelty-related LC functional connectivity and longitudinal cognitive performance on the subtests of the PACC5, as well as the executive function and memory composite scores. Analyses relating NvR LC FC, time and longitudinal cognitive scores (PACC5, PACC5 subtests, executive function composite score and memory composite score). The analyses were performed using mixed-effects models including PACC5 as outcome variable, NvR LC-FC, time, their interactions, age, sex and years of education as fixed effects, random intercepts for participants and slopes for time (number of years between baseline and follow-up cognitive assessments; number of participants n = 128 and number of observations is 753). All *p*-values were two-sided and adjusted using FDR. * Random effects were modeled using only a random intercept for each subject. Reported estimates are unstandardized. Abbreviations: Free and Cued Selective Reminder Test (FCSRT) and Preclinical Alzheimer Cognitive Composite (PACC5).

**Cognitive test**	***B***	**Standard Error**	**DF**	***t*-value**	***p*-value**	**Lower 95% CI**	**Upper 95% CI**
PACC5	4.86	0.65	621	7.45	**<0.001**	3.59	6.13
Digit symbol substitution test (DSST)	29.08	6.42	620	4.53	**<0.001**	16.56	41.60
Free recall element of FCSRT	27.99	6.15	604	4.55	**<0.001**	16.00	39.98
Total recall element of FCSRT *	16.56	1.67	604	9.94	**<0.001**	13.32	19.81
Delayed recall of the Logical Memory Test	10.85	3.37	620	3.22	**<0.001**	4.28	17.41
Mini-Mental State Exam (MMSE)	8.23	1.45	621	5.70	**<0.001**	5.42	11.05
Cognitive Abilities Test (CAT) *	27.28	4.72	621	5.78	**<0.001**	18.07	36.49
Executive composite	1.87	0.41	617	4.59	**<0.001**	1.08	2.66
Memory composite	3.04	0.76	621	4.01	**<0.001**	1.56	4.51

Supplementary Table 7: Associations between novelty-related LC functional connectivity and longitudinal PiB-related cognitive performance on the subtests of the PACC5, as well as executive function and memory scores. Analyses relating NvR LC FC, PiB, time and longitudinal cognitive scores (PACC5, PACC5 subtests, executive function composite score and memory composite score. The analyses were performed using mixed-effects models including PACC5 as outcome variable, NvR LC-FC, time, PiB, their interactions, age, sex and years of education as fixed effects, random intercepts for participants and slopes for time (number of years between baseline and follow-up cognitive assessments; number of participants n = 128 and number of observations is 753). All *p*-values were two-sided and adjusted using FDR. * Random effects were modeled using only a random intercept for each subject. Reported estimates are unstandardized. Abbreviations: Free and Cued Selective Reminder Test (FCSRT) and Preclinical Alzheimer Cognitive Composite (PACC5).

Cluster	Voxels	*P*	Max X (mm)	Max Y (mm)	Max Z (mm)	Region	Probability
1	3489	<0.001	-38	-52	-20	Temporal Fusiform Cortex	0.7
2	2566	<0.001	44	-64	-14	Lateral Occipital Cortex	0.32
						Occipital Fusiform Gyrus	0.27
3	43	<0.001	0	-34	-46	Brainstem	1

Supplementary Table 8: 3D positions of the local maxima in significant NvR brain activity regions. The reported peak voxel coordinates correspond to the 2-mm MNI152 standard-space. Only clusters with extent size greater than 40 voxels are reported. Cluster-wise *p*-values were obtained using Gaussian Random Field (GRF; number of participants n = 128; cluster defining threshold $Z > 4.5$, two-tailed $p < 0.05$, FWER-corrected). For each cluster, the brain region label and the corresponding probability of overlapping with that region according to the Harvard-Oxford brain atlas are also provided.

Cluster	Voxels	*P*	Max X (mm)	Max Y (mm)	Max Z (mm)	Region	Probability
1	1105	<0.001	-20	-10	-14	Left Amygdala	0.85
2	963	<0.001	20	-12	-14	Right Amygdala	0.58
						Right Hippocampus	0.25

Supplementary Table 9: 3D positions of the local maxima in significant NvR LC-MTL functional connectivity regions. The reported peak voxel coordinates correspond to the 2-mm MNI152 standard-space. Only clusters with extent size greater than 40 voxels are reported. Cluster-wise p-values were obtained using GRF (number of participants $n = 128$; cluster defining threshold $Z > 4.5$, two-tailed $p < 0.05$, FWER-corrected). For each cluster, the brain region label and the corresponding probability of overlapping with that region according to the Harvard-Oxford brain atlas are also provided.

Supplementary results 1: Spatial filter volume calculations

The ellipsoid Gaussian kernel employed in our study was constructed using σ = 1.125 for the major diameter and σ = 0.6. The relationship between the standard deviation of a Gaussian kernel and FWHM (in mm) is approximately given by

$$\text{FWHM} = \left(\sigma\sqrt{8\ln 2}\right) * \left(\text{voxel size (in mm)}\right).$$

Using this approximation, the major diameter is 13.24 mm (FWHM), and the minor is 4.23 mm (FWHM).

The volume of an ellipsoid with these dimensions for the major and minor diameters is given by

$$\frac{4}{3}\cdot\pi\cdot r_1\cdot r_2\cdot r_3 = \frac{4}{3}\cdot\pi\cdot 6.62\cdot 2.115\cdot 2.115 \approx 124\, mm^3,$$

where r denotes the radius of each diameter.

The volume of a spherical Gaussian filter with a diameter of 6 mm FWHM, which is frequently used in the literature [1, 2, 3, 4, 5] is given by

$$\frac{4}{3}\cdot\pi\cdot r^3 = \frac{4}{3}\cdot\pi\cdot 3^3 \approx 113\, mm^3,$$

where r denotes the radius diameter. For a spherical Gaussian filter with a diameter of 8 mm FWHM the volume is approximately equal to 268.1 mm^3, which is nearly 2 times larger than the resolution of our data after preprocessing.

Supplementary results 2: Measuring activation level using region- and condition-dependent HRFs

The analysis for quantifying the strength of the BOLD response to novel and repeated events was performed in two steps: (i) modeling of the underlying BOLD signal dynamics and the HRF associated with each experimental condition in different ROIs or voxels, and (ii) using the z-transformed model prediction output associated with each experimental condition described by equation (1) in a GLM to obtain parameter estimates (PEs) quantifying the strength of the hemodynamic response to novelty and repetition, respectively. These estimates could not be computed directly during the first step of the analysis nor extracted from the estimated HRF shapes.

The PEs obtained from a GLM analysis are more suitable measures of the strength of the hemodynamic response to novelty or repetition events compared to features (e.g area under the curve (AUC)) extracted from the estimated HRF for each condition for the following reasons:

1. The PE obtained using GLM analysis uses information associated with the entire shape of the HRF rather than information isolated in individual time-points, such as the HRF peak and time-to-peak.
2. The physiological interpretation of features extracted from the HRF (peak value, time-to-peak, power, AUC) is not straightforward [6]. Moreover, some of these features are possibly also related with properties of the vasculature rather than the underlying response to neuronal activation, such as vascular elastance and compliance [7, 8].
3. The GLM framework makes our analysis compatible with the vast majority of similar fMRI studies in the literature.

In relation to point 1, a HRF feature that might be more associated with the entire HRF curve is the area under the curve (AUC). However, AUC corresponds to the steady-state step-response to a step change [1] in neuronal activation (Supplementary Fig. 25a), which is different than the time-course of our task (mixed event-related and block design). In addition, it is often the case that HRF curves with different amplitude or shape characteristics have a similar AUC. For these reasons, we believe that AUC is not a suitable measure of the strength of the hemodynamic response to neuronal activation within the context of this study.

To illustrate this more clearly, consider a hypothetical experiment during which a subject is presented with two novelty events of which the timings are shown in Supplementary Fig. 25. A hypothetical BOLD response in a task relevant region (ROI 1 - Supplementary Fig. 25c) and a task irrelevant region (ROI 2 – Supplementary Fig. 25c) are shown in Supplementary Fig. 25d, where the amplitude of the BOLD response in ROI 1 is four times greater than in ROI 2. The HRF estimates obtained using the BOLD signal responses in each ROI and the event timings are shown

[1] The relationship of the HRF AUC and the steady-state response to step changes in neuronal activation stems from the fact that the integral of the impulse response of a linear, time-invariant system is equivalent to the system's steady-state response to a unit step function.

in Supplementary Fig. 25e. In this case, the shape of the HRF curve is the same for both ROIs. The AUC is equal to 0 for both ROIs, even though the BOLD response in ROI 1 is four times greater than in ROI 2. In contrast to this, using the GLM framework as described in the manuscript gives a parameter estimate for ROI 1 that is four times greater than the parameter estimate for ROI 2.

This example illustrates how the GLM analysis approach is more advantageous for quantifying the strength of the hemodynamic response compared to the AUC extracted from the estimated HRF.

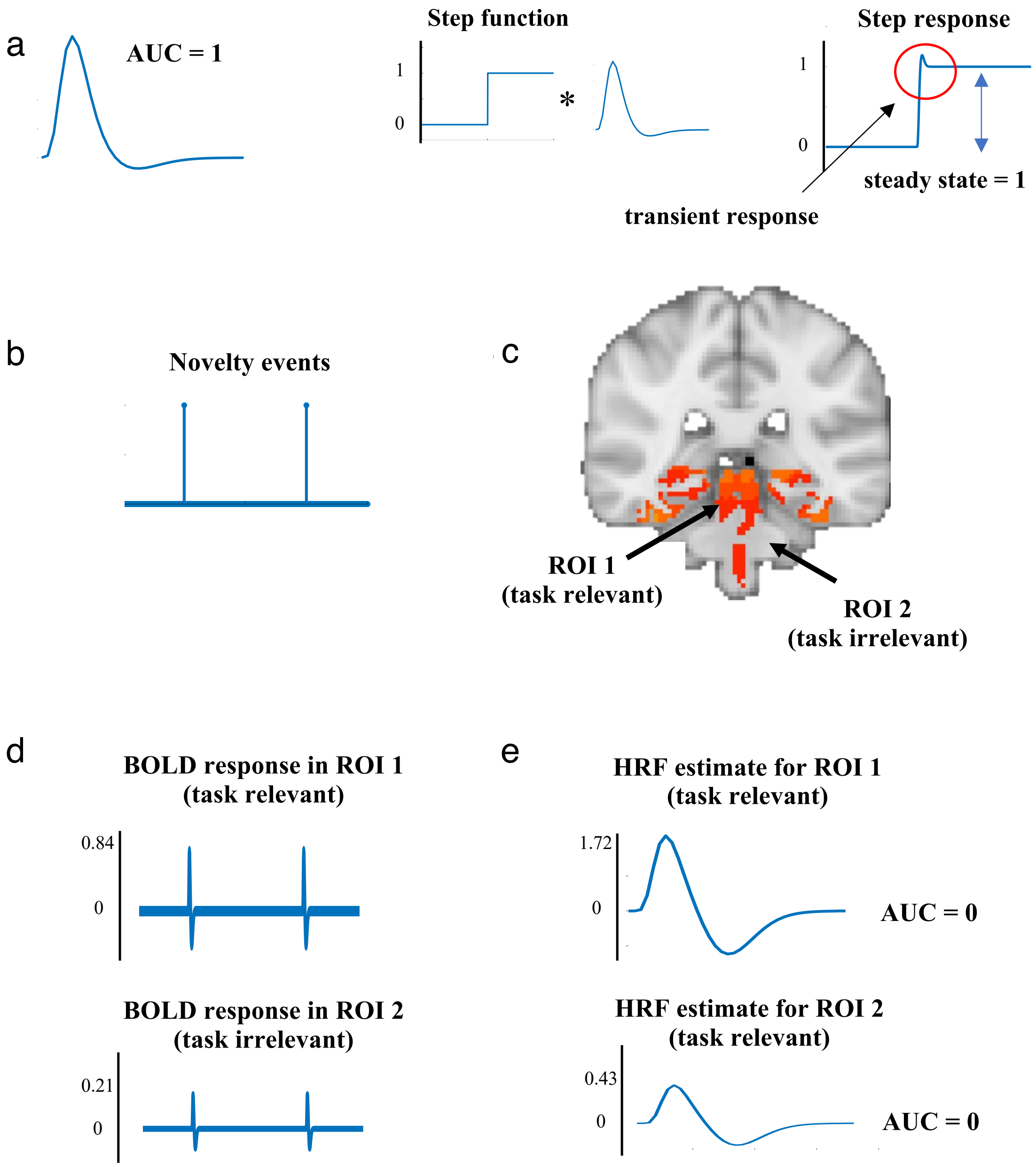

Supplementary Fig. 25: Hypothetical fMRI experiment illustrating a case when the HRF area under the curve (AUC) incorrectly indicates the absence of a significant difference in the BOLD signal response between different brain regions. (a) Illustration of the relationship between the AUC of the HRF and the steady-state step response of a linear hemodynamic model. The step response is obtained as the convolution (as indicated by *) of the hemodynamic response function with a step function and consist of a transient response followed by a steady-state response. (b) Timings of novelty events presented to a subject during a hypothetical event-related fMRI experiment. (c) Hypothetical brain activation map, where ROI 1 corresponds to a task

relevant region and ROI 2 to a task irrelevant region. (d) Hypothetical BOLD responses to the novelty events measured in ROI 1 (top) and ROI 2 (bottom). (e) HRF estimates for ROI 1 (top) and ROI 2 (bottom) obtained using the hypothetical BOLD responses shown in (e) and the timings of the novelty events in (b).

References

1. Del Cerro I*, et al.* Disrupted functional connectivity of the locus coeruleus in healthy adults with parental history of Alzheimer's disease. *J Psychiatr Res* **123**, 81-88 (2020).

2. Grueschow M*, et al.* Real-world stress resilience is associated with the responsivity of the locus coeruleus. *Nat Commun* **12**, 2275 (2021).

3. Munn BR, Müller EJ, Wainstein G, Shine JM. The ascending arousal system shapes neural dynamics to mediate awareness of cognitive states. *Nat Commun* **12**, 6016 (2021).

4. von der Gablentz J, Tempelmann C, Münte TF, Heldmann M. Performance monitoring and behavioral adaptation during task switching: an fMRI study. *Neuroscience* **285**, 227-235 (2015).

5. Yebra M*, et al.* Action boosts episodic memory encoding in humans via engagement of a noradrenergic system. *Nat Commun* **10**, 3534 (2019).

6. Lindquist MA, Meng Loh J, Atlas LY, Wager TD. Modeling the hemodynamic response function in fMRI: efficiency, bias and mis-modeling. *Neuroimage* **45**, S187-198 (2009).

7. Poublanc J*, et al.* Measuring cerebrovascular reactivity: the dynamic response to a step hypercapnic stimulus. *J Cereb Blood Flow Metab* **35**, 1746-1756 (2015).

8. Prokopiou PC, Pattinson KTS, Wise RG, Mitsis GD. Modeling of dynamic cerebrovascular reactivity to spontaneous and externally induced CO(2) fluctuations in the human brain using BOLD-fMRI. *Neuroimage* **186**, 533-548 (2019).
