# Supplementary material for: Lower novelty-related locus coeruleus function is associated with Aβ-related cognitive decline in clinically healthy individuals
Source: Nat Commun. 2022 Mar 23;13:1571. doi: 10.1038/s41467-022-28986-2 (PMC8943159; doi:10.1038/s41467-022-28986-2)
Supplement: Supplementary file 2 — Reporting Summary [file 41467_2022_28986_MOESM2_ESM.pdf]

nature research

Corresponding author(s): Dr. Heidi Jacobs

Last updated by author(s): Feb 5, 2022

Reporting Summary

Nature Research wishes to improve the reproducibility of the work that we publish. This form provides structure for consistency and transparency in reporting. For further information on Nature Research policies, see our Editorial Policies and the Editorial Policy Checklist.

Statistics

For all statistical analyses, confirm that the following items are present in the figure legend, table legend, main text, or Methods section.

n/a	Confirmed	
☐	☒	The exact sample size (n) for each experimental group/condition, given as a discrete number and unit of measurement
☐	☒	A statement on whether measurements were taken from distinct samples or whether the same sample was measured repeatedly
☐	☒	The statistical test(s) used AND whether they are one- or two-sided<br>*Only common tests should be described solely by name; describe more complex techniques in the Methods section.*
☐	☒	A description of all covariates tested
☐	☒	A description of any assumptions or corrections, such as tests of normality and adjustment for multiple comparisons
☐	☒	A full description of the statistical parameters including central tendency (e.g. means) or other basic estimates (e.g. regression coefficient) AND variation (e.g. standard deviation) or associated estimates of uncertainty (e.g. confidence intervals)
☐	☒	For null hypothesis testing, the test statistic (e.g. F, t, r) with confidence intervals, effect sizes, degrees of freedom and P value noted<br>*Give P values as exact values whenever suitable.*
☒	☐	For Bayesian analysis, information on the choice of priors and Markov chain Monte Carlo settings
☒	☐	For hierarchical and complex designs, identification of the appropriate level for tests and full reporting of outcomes
☐	☒	Estimates of effect sizes (e.g. Cohen's d, Pearson's r), indicating how they were calculated

Our web collection on statistics for biologists contains articles on many of the points above.

Software and code

Policy information about availability of computer code

Data collection	MRI data were collected with a 3T Siemens MAGNETOM Trio system (syngo MR B17). The visual presentation of the tasks stimuli was generated using an in-house Java code (Java v.1.8).
Data analysis	Data analysis scripts were written in bash and MATLAB for the MRI and PET analyses (making use of FreeSurfer version 6, ANTs version 2.1.0, FSL version 5.0.7, SPM12, MATLAB2018b), as described in sections MRI data acquisition and pre-processing, fMRI data analysis and generalized psychophysiological interaction (gPPI) fMRI analyses for the MRI data, and section PET data acquisition and pre-processing for the PET data. Statistical analyses were done in R (version 4.0.1) as described in section Statistical analyses. The software used in data analysis are also stated in the Code availability section.

For manuscripts utilizing custom algorithms or software that are central to the research but not yet described in published literature, software must be made available to editors and reviewers. We strongly encourage code deposition in a community repository (e.g. GitHub). See the Nature Research guidelines for submitting code & software for further information.

Data

Policy information about availability of data

All manuscripts must include a data availability statement. This statement should provide the following information, where applicable:

- Accession codes, unique identifiers, or web links for publicly available datasets
- A list of figures that have associated raw data
- A description of any restrictions on data availability

The Harvard Aging Brain Study project is committed to publicly releasing its data. Baseline and follow-up data until year 5 is publicly available to the research community at http://nmr.mgh.harvard.edu/lab/harvardagingbrain/data. Requests for material, data, and correspondence can be addressed to Dr. Sperling. Qualified investigators must abide by the Harvard Aging Brain Study online data use agreement, designed to protect the privacy of our participants.

Field-specific reporting

Please select the one below that is the best fit for your research. If you are not sure, read the appropriate sections before making your selection.

☒ Life sciences ☐ Behavioural & social sciences ☐ Ecological, evolutionary & environmental sciences

For a reference copy of the document with all sections, see nature.com/documents/nr-reporting-summary-flat.pdf

Life sciences study design

All studies must disclose on these points even when the disclosure is negative.

Sample size	There is no justification of sample size. This is a longitudinal cohort (panel) study and this could not be predicted beforehand. Only participants that engaged in 6 functional MRI runs of the task and had a PiB-PET scan at baseline were included (N=128 at baseline and up to 10 years follow-up measurements). However, taking into account previous work in the literature, we were convinced that our sample size would be adequate to achieve the aims of our study. Previous studies:<br>- Sperling et al., 2018 (PMID: 30549303) investigated the association and interaction of Aβ and tau on prospective cognitive decline in normal aging and preclinical Alzheimer's disease (N=137).<br>- Pihlajamäki et al., 2011 (PMID: 21161449) using the same task showed that greater MTL repeated activity was correlated with worse word-list delayed recall performance (N=60).<br>- Clewett et al., 2014 (PMID: 24667494) showed that noradrenergic influences help facilitate memory encoding during outcome processing using PPI analysis and 3T-fMRI data (N=21).
Data exclusions	No data points were excluded. For the calculation of PACC5, we allowed at most one missing subtest. Missing subtests were excluded from the calculation.
Replication	To validate our imaging results, we analyzed two different fMRI datasets. The first one, the Replication Dataset, consisted of fMRI data acquired from forty-one older individuals using an alternative version of the face-name associative task. Twenty-four individuals overlapped with the main cohort but were scanned four years later using an alternative version of the face-name associative task. The other seventeen participants joined HABS later in the study and their baseline imaging and cognitive measurements were not within one year from each other and were therefore excluded from the main sample. The characteristics of the Replication Dataset are provided in Table S1. The other dataset, the Matched Dataset, consisted of a subset of 36 Aβ- individuals who were matched to the 36 Aβ+ individuals based on the age, sex and years of education distributions using propensity-based matching. The characteristics of the Matched Dataset are provided in Table S2. Our results were further validated using additional sensitivity analyses using unsmoothed data (providing the highest spatial resolution), an eroded version of the LC ROI (for the gPPI analyses), as well as grey matter density as a covariate. Each of these validation and sensitivity analyses reproduced our imaging results.
Randomization	N/A. There was no intervention.
Blinding	All investigators (clinicians) were blind to biomarker /genetics status and imagers were blind to biomarker/APOE status and cognitive performance.

Reporting for specific materials, systems and methods

We require information from authors about some types of materials, experimental systems and methods used in many studies. Here, indicate whether each material, system or method listed is relevant to your study. If you are not sure if a list item applies to your research, read the appropriate section before selecting a response.

Materials & experimental systems

n/a	Involved in the study
☒	☐ Antibodies
☒	☐ Eukaryotic cell lines
☒	☐ Palaeontology and archaeology
☒	☐ Animals and other organisms
☐	☒ Human research participants
☒	☐ Clinical data
☒	☐ Dual use research of concern

Methods

n/a	Involved in the study
☒	☐ ChIP-seq
☒	☐ Flow cytometry
☐	☒ MRI-based neuroimaging

Human research participants

Policy information about studies involving human research participants

Population characteristics	One hundred twenty-eight older individuals from the Harvard Aging Brain Study underwent imaging, as well as longitudinal neuropsychological evaluations over up to 10 years. Seventy-one participants (55.46%) were female. At baseline, the mean age of the participants was 70.07 ± 8.86 (SD) and the mean education level was 15.74 ± 2.67 (SD) years. In addition, all participants had no history of medical or psychiatric disorders and were clinically unimpaired at baseline: Mini-Mental State Examination (MMSE) > 25 and Clinical Dementia Rating (CDR) = 0. Thirty-six participants (28.1%) were classified as Aβ positive. This information is also stated in Table 1, which also provides an overview of the differences between Aβ positive and negative individuals.
Recruitment	Participants were recruited from a longitudinal cohort followed at the Alzheimer Disease Research Center (ADRC) at Massachusetts General Hospital (MGH). In addition, participants were recruited through advertisements in local newspapers, internet sites and community-based outreach events. The Harvard Aging Brain Study has recruited more white and highly educated individuals than expected based on the New England population, therefore results may be less generalizable to other communities.
Ethics oversight	The study complied with all ethical regulations and was approved by the MGB/Partners Human Research Committee at Massachusetts General Hospital. All participants provided written informed consent following the MGB/Partners Human Research Committee regulations, and received monetary compensation after each visit.

Note that full information on the approval of the study protocol must also be provided in the manuscript.

Magnetic resonance imaging

Experimental design

Design type	Task (encoding of face-name associations); Mixed block/event-related design.
Design specifications	The task comprised events of unfamiliar and familiar face-name pairs organized within blocks of novelty and repetition, respectively. The novelty blocks consisted of 7 face-name pairs (Ni, i=1,..7). The repetition blocks consisted of 7 trials during which two face-name pairs were alternated, one male and one female. (Rj, j=1,2). The novelty, repetition and visual fixation (+) blocks, as well as the events within the blocks (Ni, i=1,...7; Rj, j=1,2; +) are depicted along with their corresponding duration. Each block was shown twice and alternated with visual fixation blocks. One functional run lasted for 4 minutes and 5 seconds, and a total of 6 functional runs were presented to each participant.
Behavioral performance measures	N/A. No behavioral performance measures were used in this study.

Acquisition

Imaging type(s)	Structural, functional
Field strength	3T
Sequence & imaging parameters	T1-weighted MPRAGE sequence: TR/TE = 2300/2.95 ms; Voxel size = 1.1. x 1.1 x 1.2 mm; Flip Angle = 90°; Number of slices = 176; Acquisition matrix = 270 x 254 x 212 mm; Orientation = sagittal; Inversion time = 900 ms; 2X (GRAPPA) acceleration.<br><br>T2*- weighted EPI sequence: TR/TE = 2000/30 ms; Voxel size = 3.1 x 3.1 x 5.0 mm; Flip Angle = 90°; Number of slices = 30; Acquisition matrix = 200 x 200 x 179 mm; Field of View read = 200 mm; Orientation = coronal.
Area of acquisition	Whole brain acquisition

Diffusion MRI ☐ Used ☒ Not used

Preprocessing

Preprocessing software	FSL version 5.0.7: brain extraction, slice timing correction, motion correction via volume realignment, normalization to the 2 mm isotropic MNI-152 EPI template. Spatial smoothing was performed using a custom ellipsoid Gaussian kernel.
Normalization	We initially aligned the BOLD images to the high resolution 1 mm - T1 structural image of each subject using boundary-based registration (BBR). Subsequently, the T1 structural image was aligned with the MNI-152 template using a 3-step registration procedure: in the first step the T1 structural image was registered to the MNI-152 template using an affine, linear registration with 12 degrees of freedom. In the second step, this affine registration was refined using cost-function weighting input and reference volumes. The input and reference weighting images are provided in the Supplementary Material. In the third step, a non-linear registration was performed, which was initialized using the transformation matrix obtained from the previous step.
Normalization template	MNI-152 (2 mm isotropic)
Noise and artifact removal	Noise and artifact removal was performed using ICA-AROMA and nuisance regression. The nuisance regressors included

three ROI time-series obtained as the mean across voxels in the 4th ventricle, lateral ventricles and white matter, the 6 motion parameters (MP) generated during volume realignment, the derivatives of the 6 MPs and the squares of all the aforementioned time-series.

Volume censoring	Detection and removal of motion-contaminated volumes was performed based upon the derivative of root mean squared variance over voxels (DVARS), which was estimated in FSL using the boxplot threshold (the 75th percentile + 1.5 times the interquartile range).

Statistical modeling & inference

Model type and settings	We performed both voxelwise and ROI-based analyses. First level analysis (voxelwise): general linear model (GLM) analysis using one constant term for modeling the intercept, and two regressors each of which were associated with a different experimental condition. The time-course of each condition was convolved with a condition-dependent HRF curve that was estimated directly from the data using a function expansion technique. For the gPPI analysis, we also included one physiological and two interaction regressors. Subsequently, the derived regressors were z-transformed and entered into a GLM analysis. Temporal auto-correlation was modeled using a sixth-order auto-regressive sequence. A parameter estimate for each regressor was obtained using ordinary least squares regression. The two conditions (for the NvR brain activation analysis; see Figure 3) or the interaction regressors (for the gPPI analysis; see Figure 4) were contrasted against each other to create contrast images of Novel versus Repeated Face-Name Pairs (NvR). Second level (group level): statistical parametric maps were generated using the subject-specific NvR contrast images using linear-mixed effects modeling, which included baseline age and sex as covariates, random intercepts for subjects and slopes for functional runs. The second level analysis was also performed in ROIs using extracted subject-specific NvR LC activity and LC-MTL FC values. This information is also provided in sections fMRI data analysis, Generalized psychophysiological interaction (gPPI) fMRI analyses, and Statistical analyses.
Effect(s) tested	Detection of NvR brain activity and LC-MTL FC were performed cross-sectionally. Associations between LC activity or LC-MTL FC with Aβ-related cognitive decline were assessed cross-sectionally and longitudinally. No ANOVA or factorial designs were used, but linear regression models and mixed effects models were performed for cross-sectional and longitudinal analyses, respectively. See section Statistical Analyses.

Specify type of analysis: ☐ Whole brain ☐ ROI-based ☒ Both

Anatomical location(s)	Structurally defined ROIs were obtained using an automated labeling algorithm in FreeSurfer (see Figure S23 and section MRI data acquisition and pre-processing). Functionally defined ROIs were defined based on regions exhibiting significant associations between LC activity or LC-MTL FC and cognitive decline (see Figure 5 and Figure 7).
Statistic type for inference (See Eklund et al. 2016)	Cluster-wise methods using cluster defining threshold of either Z > 4.5 for analyses including MRI and PET data, or Z > 3.1 for analyses including MRI, PET and neuropsychological data.
Correction	FWE and FDR (two-tailed $p < 0.05$).

Models & analysis

n/a	Involved in the study
☐	☒ Functional and/or effective connectivity
☒	☐ Graph analysis
☐	☒ Multivariate modeling or predictive analysis

Functional and/or effective connectivity	Contrast values of Novel versus Repeated face-name pairs obtained using gPPI analysis.
Multivariate modeling and predictive analysis	For modeling the BOLD signal and performing HRF estimation we used finite impulse response model analysis and function expansions in terms of the spherical Laguerre basis. Model order selection was determined based on the Bayesian Information Criterion and the parameters of the spherical Laguerre basis were selected using cross-validation based on the model mean squared error (see section fMRI data analysis).
